# PVN-centered multi-omics and single-cell analyses suggest shared inflammatory and immune features in acute myocardial infarction-associated ventricular arrhythmia and atrial fibrillation

**DOI:** 10.3389/fcvm.2026.1768762

**Published:** 2026-09-03

**Authors:** Wenlong Wang, Wenbo Zhao, Yuran Chen, Xin Wang, Chunrui Ji, Jingmei Sun, Yu Bai, Yuhang Yang, Xiufen Qu

**Affiliations:** Department of Cardiology, The First Affiliated Hospital of Harbin Medical University, Harbin, China

**Keywords:** acute myocardial infarction, atrial fibrillation, bilirubin, Mendelian randomization, multi-omics, NF-κB, paraventricular nucleus, ventricular arrhythmia

## Abstract

**Objective:**

Acute myocardial infarction (AMI) increases the risk of arrhythmias, particularly ventricular arrhythmias (VA), and atrial fibrillation (AF) is also common after AMI. The paraventricular nucleus (PVN) is a key regulator of sympathetic outflow. This study used a PVN-centered strategy to investigate molecular changes after AMI, prioritize candidate molecules associated with VA, and assess whether related inflammatory and immune features are also present in AF cohorts.

**Methods:**

An AMI model was established in C57BL/6 mice, and PVN tissues were collected at 48 h and 7 d for transcriptomic and metabolomic profiling. Electrocardiogram (ECG) telemetry, histological assessment and serum markers were used to evaluate infarct injury and arrhythmia burden, and immunofluorescence was used to assess glial responses and nuclear factor-*κ*B (NF-*κ*B) activation in the PVN. Two-sample Mendelian randomization (MR), summary-data-based Mendelian randomization (SMR) and colocalization analyses were performed to prioritize candidate molecules associated with VA. External analyses were conducted in AF cohorts (GSE41177 and GSE261170). Functional support was evaluated by bilirubin intervention *in vivo* and Il12rb1 knockdown in a BV2 hypoxia/reoxygenation (H/R) model.

**Results:**

AMI increased premature ventricular contractions and ventricular tachycardia burden, together with elevated cardiac troponin I and increased ionized calcium-binding adaptor molecule 1 (Iba1), glial fibrillary acidic protein (GFAP) and phosphorylated NF-*κ*B p65 (p-p65) signals in the PVN. MR suggested an inverse association between direct bilirubin and VA risk, and bilirubin treatment reduced arrhythmia burden and PVN inflammatory activation. MR/SMR analyses prioritized DGKQ, BLM, ABCA3 and IL12RB1 as candidate genes associated with VA. In AF cohorts, these genes were differentially expressed, and pathway, immune infiltration and single-cell analyses suggested inflammatory, hypoxic and immune remodeling in cardiac tissue. *In vitro*, Il12rb1 knockdown reduced p-p65, interleukin-6 (IL-6) and tumour necrosis factor-α (TNF-α) under H/R conditions.

**Conclusion:**

These findings support a PVN-centered framework in which inflammatory and immune processes may contribute to post-AMI arrhythmia vulnerability. Direct bilirubin and DGKQ, BLM, ABCA3 and IL12RB1 may represent candidate molecules for further investigation.

## Graphical Abstract

**Figure:**
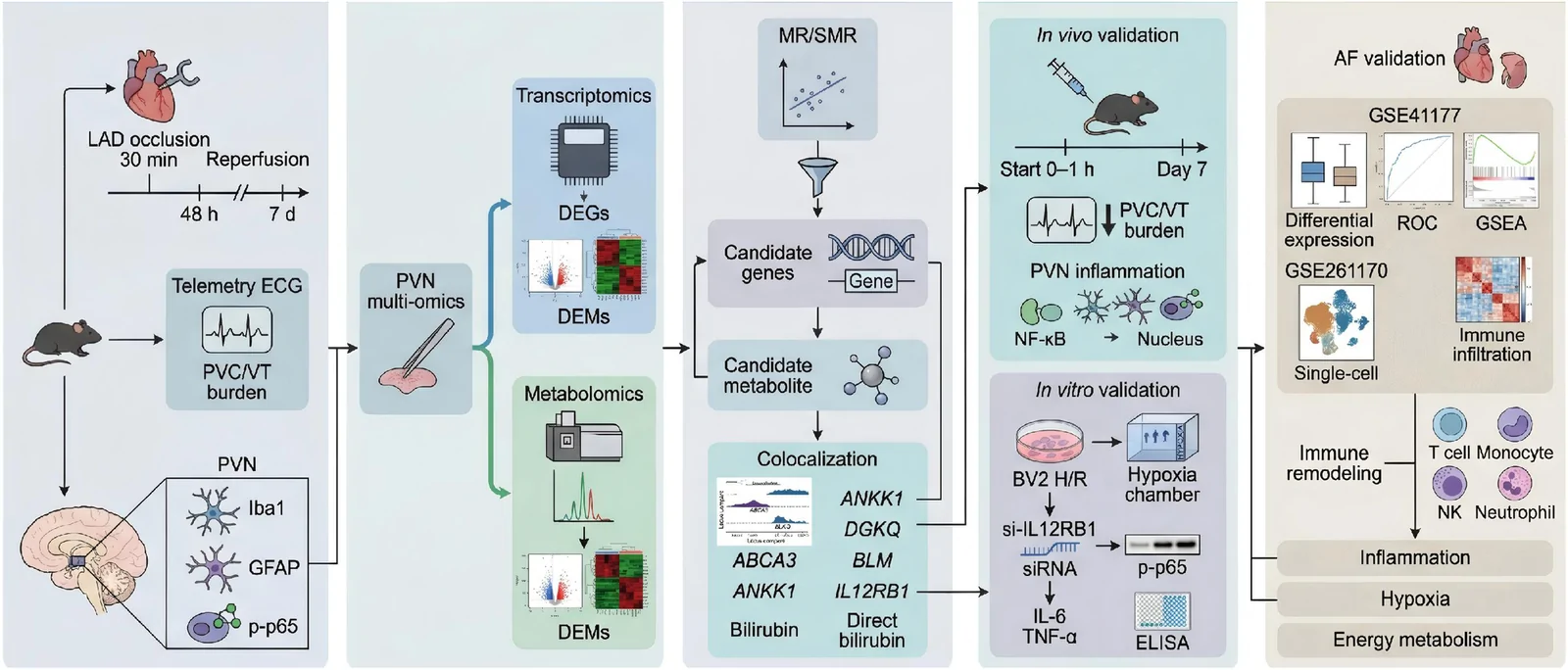

## Introduction

Acute myocardial infarction (AMI) markedly increases susceptibility to cardiac arrhythmias, particularly ventricular arrhythmias (VA), which remain an important cause of early death after infarction (1, 2). In addition to causing acute ischaemic myocardial injury (3), AMI is accompanied by inflammatory activation and autonomic imbalance, both of which are closely related to post-infarction arrhythmogenesis (2, 4). Atrial fibrillation (AF) is also frequently observed during or after AMI and is associated with adverse clinical outcomes, including heart failure, stroke and increased mortality (5–7).

The paraventricular nucleus (PVN) of the hypothalamus is a major integrative centre for sympathetic outflow. Experimental studies have shown that myocardial infarction can be followed by inflammatory and microglial activation within the PVN (8, 9), and that modulation of PVN glial or inflammatory signalling may attenuate sympathetic excitation and reduce ventricular arrhythmia susceptibility after infarction (10–12). These observations support the relevance of PVN remodelling to post-AMI arrhythmia vulnerability, but the underlying molecular changes remain incompletely defined.

VA and AF are both clinically relevant after AMI, although they are usually studied separately. VA is closely linked to acute electrical instability and sudden death, whereas AF is a frequent complication with important prognostic implications after infarction (1, 5). Despite their distinct clinical manifestations, both arrhythmias are associated with inflammatory activation, hypoxic stress and autonomic disturbance after AMI (2, 13). This provides a rationale for using VA as the primary phenotype of post-AMI arrhythmia susceptibility while also examining whether related inflammatory and immune features are detectable in independent AF datasets.

Mechanistically, post-infarction cytokine signaling can promote arrhythmia susceptibility by contributing to electrical remodeling, structural remodeling, ion-channel dysfunction, fibrosis, and autonomic dysregulation, thereby linking inflammatory activation to both VA and AF after myocardial injury (14).

Recent advances in multi-omics and genetic association analyses provide opportunities to investigate complex cardiovascular phenotypes across molecular layers. Integrative omics studies have identified candidate genes and pathways in AF and other cardiovascular disorders (15), but most previous analyses have focused on atrial tissue, myocardium, blood or peripheral autonomic structures rather than the central nervous system. As a result, three questions remain insufficiently addressed: how the PVN is molecularly remodelled after AMI, which PVN-derived candidate molecules are associated with VA risk, and whether related inflammatory and immune features are also present in human AF cohorts.

The present study therefore adopted a PVN-centred framework that combined transcriptomic and metabolomic profiling in a mouse AMI model with summary-data-based Mendelian randomization (SMR), colocalization analysis, human AF cohort analysis and experimental validation. The aims were to characterize molecular remodelling in the PVN after AMI, prioritize candidate molecules associated with VA, and assess whether related inflammatory and immune features are also present in AF cohorts. This integrative strategy was designed to provide a systems-level view of post-AMI arrhythmia vulnerability and to identify candidates for further investigation.

## Materials and methods

### Construction and intervention of the AMI animal model

Eight-week-old male C57BL/6J mice were obtained from Beijing Vital River Laboratory Animal Technology Co., Ltd. An AMI/reperfusion model was established by transient ligation of the left anterior descending coronary artery (LAD), as shown in Supplementary Figure S1. Mice were anesthetized with inhaled isoflurane, a commonly used inhalational anesthetic for murine ischemia/reperfusion models, the chest was opened, and the LAD was ligated for 30 min followed by reperfusion (16, 17). Postoperative analgesia was provided with ibuprofen at 10 mg/kg, and animals were monitored daily for body temperature, activity and food intake. To characterize arrhythmia burden and PVN inflammatory changes after AMI, animals were assigned to four groups for model phenotyping: Sham 48 h, AMI 48 h, Sham 7 d and AMI 7 d (*n* = 8 biologically independent animals per group). For bilirubin intervention, animals were assigned to Sham + Vehicle, AMI + Vehicle and AMI + Bilirubin groups and were evaluated at both 48 h and 7 d (*n* = 8 biologically independent animals per group per time point). Bilirubin was administered intraperitoneally at 30 mg/kg, with the first dose given within 1 h after surgery and repeated once daily thereafter (18, 19). At 48 h and 7 d, serum was collected for direct bilirubin and cardiac troponin I (cTnI) measurements, and PVN-containing brain tissue was harvested for immunofluorescence analysis. All experimental procedures adhered to the ethical guidelines approved by the Institutional Animal Care and Use Committee (Ethics Approval No: JSAB25007M).

### Electrocardiogram (ECG) telemetry

A mouse electrocardiogram (ECG) telemetry system was used for continuous ECG recording. To minimize implantation-related stress and obtain stable baseline signals, telemetry transmitters were implanted 7 d before AMI or sham surgery, followed by an adequate recovery period. Continuous ECG monitoring was initiated immediately after surgery, with emphasis on the acute 0–48 h postoperative window to capture peak ventricular arrhythmia (VA) incidence, and an additional continuous 24 h recording was performed on postoperative day 7 to assess arrhythmia burden during the recovery phase.

Premature ventricular contractions (PVCs) were defined as early wide QRS complexes occurring before the expected sinus beat, usually without a preceding P wave or with P–QRS dissociation, and typically followed by a compensatory pause. Ventricular tachycardia (VT) was defined as three or more consecutive PVC-like wide QRS complexes. When applicable, VT was further classified as nonsustained or sustained according to predefined duration thresholds. Arrhythmia burden was quantified as PVC burden (PVCs/h), VT episode count, and total VT duration (s).

### TTC (2,3,5-Triphenyltetrazolium chloride) staining

To verify successful establishment of the myocardial infarction model, mice were euthanized at 48 h or 7 d after surgery. Hearts were rapidly excised, rinsed thoroughly with ice-cold saline, and cut transversely into consecutive slices approximately 1 mm thick. The slices were then incubated in 1% 2,3,5-triphenyltetrazolium chloride (TTC) solution (Sigma-Aldrich, T8877) at 37 °C for 15–20 min in the dark. After staining, the sections were fixed in 4% paraformaldehyde and photographed. Viable myocardium was stained red, whereas infarcted regions remained white or pale yellow. Infarct size was quantified using ImageJ software as the percentage of infarct area relative to the total left ventricular area.

### PVN RNA sequencing

PVN-containing coronal brain sections were identified according to a mouse brain atlas, and the PVN region was microdissected on ice. Total RNA was extracted from PVN tissue using TRIzol reagent (Invitrogen, 15596018), and RNA integrity was assessed using an Agilent 2100 Bioanalyzer (Agilent Technologies, USA). Only samples with an RNA integrity number (RIN) >= 7.0 and without obvious degradation were used for sequencing. RNA-seq libraries were prepared using the NEBNext Ultra II RNA Library Prep Kit for Illumina (New England Biolabs, E7770) according to the manufacturer's instructions and sequenced on an Illumina NovaSeq 6,000 platform with a paired-end 150-bp read configuration. Raw reads were subjected to adaptor trimming and quality filtering, and clean reads were aligned to the mouse reference genome using STAR (version 2.7.10a). Gene-level read counts were generated using featureCounts (Subread version 2.0.3). Read count matrices were imported into R for downstream analysis. Lowly expressed genes were filtered before normalization and differential expression analysis.

### PVN metabolomics

For metabolomic profiling, PVN tissue was homogenized in a pre-cooled organic extraction solvent and centrifuged at 4 ℃ to remove precipitated proteins. The supernatants were subjected to liquid chromatography–mass spectrometry (LC-MS)-based untargeted metabolomic analysis in positive and/or negative ionization modes. Raw data were processed for peak detection, alignment and peak integration, and metabolite features with excessive missing values were removed. Signal drift or batch effects were corrected where necessary, followed by data normalization. Metabolite annotation was performed by matching accurate mass, retention time and/or MS/MS spectra against available metabolite databases. When partial least squares discriminant analysis (PLS-DA) was performed, variable importance in projection values were calculated to assist feature prioritization.

### Construction and intervention of the hypoxia/reoxygenation (H/R) model

BV2 microglial cells were cultured in Dulbecco's modified Eagle medium (DMEM) supplemented with 10% fetal bovine serum (FBS) and 1% penicillin-streptomycin at 37 °C in a humidified atmosphere containing 5% CO2. For construction of the hypoxia/reoxygenation (H/R) model, cells were exposed to 1% O2 for 4 h and then returned to normoxic conditions for 24 h of reoxygenation. Cells were transfected with negative-control siRNA (siNC) or Il12rb1-targeting siRNA (siIl12rb1) using Lipofectamine 3,000 transfection reagent (Invitrogen, L3000015) according to the manufacturer's protocol. Two experimental groups were established: H/R + siNC and H/R + siIl12rb1. Each experiment was independently repeated three times (*n* = 3 biological replicates).

Real-time quantitative PCR (qRT-PCR) was performed to verify Il12rb1 knockdown efficiency and to quantify Il-6 and Tnf mRNA expression. Total RNA was extracted from BV2 cells using TRIzol reagent, reverse-transcribed into cDNA using a PrimeScript RT reagent kit (Takara, RR047A), and amplified using TB Green Premix Ex Taq II (Takara, RR820A) on a QuantStudio 6 Flex Real-Time PCR System (Applied Biosystems). Relative expression levels were calculated using the 2^-ΔΔCt method with Gapdh as the internal reference gene.

For western blot analysis, total cellular protein was extracted after H/R treatment, separated by SDS-PAGE, transferred onto polyvinylidene difluoride membranes, and incubated with primary antibodies against phosphorylated NF-*κ*B p65 (p-p65) and the corresponding loading control overnight at 4 °C. After incubation with horseradish peroxidase-conjugated secondary antibodies, immunoreactive bands were detected using enhanced chemiluminescence and quantified by densitometry with ImageJ software.

### Enzyme-linked immunosorbent assay (ELISA)

Postoperative blood samples were collected via retro-orbital bleeding, centrifuged at 3,000 x g for 10 min at 4 °C, and the supernatant serum was harvested and stored at −80 °C until analysis. Serum bilirubin was measured using a commercial colorimetric assay kit for total and direct bilirubin (Abcam, ab235627). Serum cardiac troponin I (cTnI) was measured using a mouse cardiac troponin I ELISA kit (Abcam, ab246529) according to the manufacturer's instructions. Absorbance was measured at 450 nm using a microplate reader, and analyte concentrations were calculated from standard curves.

After completion of H/R treatment, culture supernatants were collected and centrifuged to remove cellular debris. The concentrations of interleukin-6 (IL-6) and tumor necrosis factor-alpha (TNF-alpha) were measured using commercial ELISA kits (Abcam; catalog numbers: ab178013 and ab181421) according to the manufacturers' instructions.

### Immunofluorescence staining

Brains were fixed in 4% paraformaldehyde, dehydrated in sucrose solution, and embedded for frozen sectioning. Coronal sections containing the paraventricular nucleus (PVN) were identified according to a mouse brain atlas and prepared as frozen sections (20 μm). After permeabilization with 0.3% Triton X-100 and serum blocking, sections were incubated overnight at 4 °C with primary antibodies against Iba1, glial fibrillary acidic protein (GFAP), and phosphorylated NF-*κ*B p65 (p-p65). On the following day, sections were incubated with fluorescent secondary antibodies, counterstained with DAPI, and mounted. Immunofluorescence images were acquired using a confocal laser scanning microscope (Leica TCS SP8, Leica Microsystems), and the same laser power, exposure time, gain, and imaging settings were applied across all comparable sections.

For quantitative immunofluorescence analysis, a fixed number of visual fields were captured from anatomically matched PVN sections for each animal, and the mean value across fields was treated as one biological replicate. Iba1 signals were quantified as the density of Iba1-positive cells (cells/mm^2^), GFAP signals were quantified as the percentage of positive area (% area), and p-p65 signals were quantified as the proportion of p-p65-positive cells (%). Image quantification was performed using ImageJ software (National Institutes of Health, USA).

### Data acquisition and preparation

Self-generated data comprised PVN transcriptomic and metabolomic profiles from sham and AMI mice collected at 48 h and 7 d after surgery.

The human AF microarray dataset GSE41177 was downloaded from the Gene Expression Omnibus (GEO) using GEOquery (version 2.70.0) in R (version 4.2.2). According to the GEO record and the original publication, this dataset contains paired left atrial-pulmonary vein junction (LA-PV junction) and left atrial appendage (LAA) specimens from 16 patients with persistent AF undergoing valvular surgery and 3 patients with sinus rhythm undergoing valvular surgery, yielding a total of 38 microarray samples on the GPL570/Affymetrix Human Genome U133 Plus 2.0 Array platform. Because each subject contributed two anatomically distinct but paired left atrial samples, the analysis accounted for within-subject pairing by including patient identity as a blocking factor and tissue region as a covariate, thereby reducing bias from treating paired samples as fully independent observations. Controls were therefore valvular-disease patients in sinus rhythm rather than healthy donors. Raw CEL files were processed by robust multi-array average (RMA) normalization, and outlier samples were assessed by principal component analysis (PCA) and hierarchical clustering before downstream analysis. Detailed information is provided in Table 1 (20).

**Table 1:** GEO microarray chip information.

GEO accession	GSE41177
Platform	GPL570
Species	Homo sapiens
Tissue	Heart
Samples in AF group	32
Samples in Control group	6
Reference	(20)

GEO, Gene Expression Omnibus; AF, Atrial fibrillation.

Blood cis-expression quantitative trait locus (cis-eQTL) summary statistics were obtained from eQTLGen phase I, which includes up to 31,684 blood and peripheral blood mononuclear cell samples. Variants associated with gene expression at *P* < 5 × 10^-8 were considered candidate instruments. Ventricular arrhythmia (VA) outcome summary statistics were obtained from the IEU OpenGWAS resource using the study identifier ebi-a-GCST90018940. There was no substantial sample overlap between the eQTLGen and VA GWAS datasets, thereby reducing the risk of weak-instrument bias caused by sample overlap (21).

The single-cell RNA-sequencing dataset GSE261170 was analyzed based on the original publication reporting left atrial single-cell transcriptomics in atrial fibrillation. In that study, AF left atrial samples were generated by the authors, whereas healthy control samples were obtained from the Human Cell Atlas Data Coordination Platform (ERP12313814). The count matrix was imported into Seurat (version 5.0) using the CreateSeuratObject function, and samples were integrated using IntegrateData after standard preprocessing and quality control (22). Low-quality cells were excluded on the basis of nFeature_RNA, nCount_RNA, and percent.mt. Potential doublets were identified using the DoubletFinder (version 2.0.6) algorithm. After quality control, the data were normalized, scaled, and subjected to dimensionality reduction by principal component analysis. Cell clusters were identified using graph-based clustering and visualized by uniform manifold approximation and projection. Cell type annotation was performed using SingleR (version 2.12.0) with celldex (version 1.18.0) and refined according to established marker genes.

### Differential expression analysis

Differential expression analysis of genes and metabolites between AMI groups and their corresponding sham-operated groups was performed in R using limma (version 3.58.1). For the mouse PVN transcriptome, genes with |log2FC| > 0.5 and adjusted *p* value <0.05 were considered differentially expressed. For the human AF microarray dataset GSE41177, genes with |log2FC| > 1 and adjusted *p* value <0.05 were considered differentially expressed. False discovery rate correction was performed using the Benjamini-Hochberg method. For metabolomic differential analysis, variable importance in projection (VIP) values were calculated based on the PLS-DA model, and VIP > 1 was used to assist metabolite prioritization. To control for batch effects, data were adjusted using the sva package (version 3.50.0). Visualization was performed using ggplot2 (version 3.4.4) and pheatmap (version 1.0.12).

Differentially expressed genes and differentially expressed metabolites were submitted to MetaboAnalyst for joint pathway and network analysis, and the resulting gene–metabolite interaction network was visualized using Cytoscape (version 3.10.4) (23, 24).

### Mendelian randomization and SMR analysis

Two-sample Mendelian randomization (MR) analysis was performed using candidate DEMs and eQTL-derived gene-expression instruments as exposure factors and ventricular arrhythmia (VA) as the outcome variable. Instrumental variables (IVs) were selected using the following criteria: F-statistic >10, linkage disequilibrium (LD) clumping threshold of *r*^2^ < 0.001, and a distance window of 10,000 kb. For exposure factors with only one single nucleotide polymorphism (SNP), the Wald ratio method was used to evaluate MR results; for those with two or more SNPs, the inverse variance weighted (IVW) method was applied. Heterogeneity tests, horizontal pleiotropy tests, leave-one-out analyses, and Steiger's directionality test were conducted using the TwoSampleMR package (version 0.6.0) in R.

Summary-data-based Mendelian randomization (SMR) leverages GWAS summary statistics and eQTL data to test pleiotropic associations between gene expression and complex traits. SMR analysis was performed using the SMR software package (Linux version 2.3.1) with default parameters. Because the eQTL instruments were blood-derived, these analyses were interpreted as prioritizing candidate genes associated with VA rather than as tissue-specific evidence for PVN expression effects (25–27).

### Bayesian colocalization analysis

For genes with significant MR results in eQTLGen, Bayesian colocalization analysis was performed using the coloc package (version 5.2.3) in R to determine whether the association between gene expression and VA was driven by a shared causal variant at a given locus rather than by linkage disequilibrium alone. Colocalization probability was estimated for each locus using the default prior settings unless otherwise specified. Posterior probability for hypothesis 4 (PP.H4) ≥ 0.8 was considered strong colocalization, whereas 0.5 ≤ PP.H4 < 0.8 was considered moderate colocalization (28).

### Receiver operating characteristic (ROC) analysis

Receiver operating characteristic (ROC) curves for key genes were generated using the R package pROC (version 2.18.5), and the area under the curve (AUC) was calculated to assess the discriminatory performance of key gene expression levels within the GSE41177 cohort. The AUC threshold for the ROC curve is set at >0.7. A higher AUC value closer to 1 indicates better diagnostic performance. The controls in GSE41177 were valvular-disease patients in sinus rhythm rather than healthy individuals, ROC results were interpreted as within-cohort discrimination rather than as diagnostic performance in the general population (20).

### Drug prediction and molecular docking

The Drug Signature Database (DSigDB), a component of the Enrichr platform, was used for compound prediction based on the screened key genes. Specifically, the identified key genes were submitted to DSigDB to explore their associations with candidate drugs or compounds.

To further explore the potential binding patterns between key genes and their corresponding compounds, molecular structures were downloaded from the PubChem database, and x-ray crystal structures of key proteins were obtained from the Protein Data Bank (PDB). For proteins without experimentally resolved structures, such as ABCA3 and DGKQ, predicted structures were downloaded from the AlphaFold Protein Structure Database, and major domains with predicted local distance difference test (pLDDT) scores >70 were selected. Blind docking and visualization of protein–compound interactions were performed using CB-Dock2, which integrates cavity detection, docking, and homologous template fitting, based on the AutoDock Vina engine. Docking parameters were set to default values, with a grid box size of 40 × 40 × 40 Å and an exhaustiveness value of 16. The docking score (Vina score) was interpreted as follows: Vina score > −4 kcal/mol indicated very weak or no binding; −7 kcal/mol < Vina score <  −4 kcal/mol indicated moderate binding; and Vina score < −7 kcal/mol indicated strong binding affinity (29–32).

### Correlation and chromosomal localization analysis of Key genes

To further investigate the correlation among key genes, the Spearman algorithm was employed to analyze the expression levels of key genes in GSE41177. The results were visualized using chord diagrams generated by the R packages igraph (version 2.6.0) and ggraph (version 2.2.0). A correlation was considered statistically significant when the absolute value of the correlation coefficient (*r*-value) exceeded 0.3. Finally, chromosomal localization maps of the key genes were constructed using the R package RCircos (version 2.2.2).

### Gene ontology (GO) and Kyoto encyclopedia of genes and genomes (KEGG) enrichment analysis

GO analysis is a widely utilized method for functional enrichment, encompassing various aspects, including biological processes (BP), cellular components (CC), and molecular functions (MF). The KEGG database is extensively employed to store information on genomes, biological pathways, diseases, and drugs. For the analysis of key genes, the R package clusterProfiler (version 4.10.0) was used to conduct GO and KEGG enrichment analyses. The selection criteria were set at a *p*-value < 0.05 and an FDR (*q*-value) < 0.25.

### Gene Set enrichment analysis (GSEA)

GSEA is employed to evaluate the distribution trends of predefined gene sets within a list of genes ranked by phenotype correlation, thereby assessing their contribution to the phenotype. Initially, genes from GSE41177 were ranked based on log_2_FC values derived from differential analysis results. Subsequently, the R package clusterProfiler (version 4.10.0) was utilized to conduct GSEA on all genes in GSE41177. A random seed of 2020 was set, with each gene set containing a minimum of 10 and a maximum of 500 genes. The gene sets c2.all.v2023.2.Hs.symbols used in this analysis were sourced from the MSigDB database (https://www.gsea-msigdb.org/gsea/msigdb). The selection criteria were set at an adj.*p* < 0.05 and an FDR (*q*-value) < 0.25, with *p*-value correction applied using the BH method.

### Immune infiltration analysis

Immune infiltration in GSE41177 was estimated by single-sample gene set enrichment analysis (ssGSEA), which quantifies the relative abundance of immune cell types in each sample. Immune cell signature gene sets were curated based on previous research (33) and the MSigDB C7 immune gene set. The ssGSEA algorithm was applied to calculate enrichment scores for each immune cell type, thereby constructing an immune infiltration matrix for GSE41177. Enrichment scores were normalized at the sample level to ensure comparability across samples. Boxplots were generated using ggplot2 to visualize differences in immune cell abundance between control and AF groups. Immune cells showing significant differential abundance between groups were identified, and their intercellular correlations were calculated using the Spearman algorithm and visualized by heatmaps generated with pheatmap. In addition, correlations between key genes and differentially abundant immune cells were calculated using Spearman correlation and visualized using bubble plots created with ggplot2.

### Single-Cell analysis

Quality Control: The expression matrices of all samples from GSE261170 were merged to create a Seurat object using Seurat (version 5.0). The following filtering criteria were applied: genes had to be expressed in at least three cells, and each cell had to express at least 200 genes. Cells meeting any of the following conditions were excluded: total counts < 500, log10GenesPerUMI < 0.8, or features <250. Potential doublets were identified and removed using the DoubletFinder algorithm.

Normalization and Dimensionality Reduction: Sequencing depth normalization of the GSE261170 data was performed using the NormalizeData function with the “LogNormalize” method. The FindVariableFeatures function was used to identify the top 2,000 highly variable genes based on the “vst” method. The ScaleData function was then applied to standardize the data. Principal component analysis (PCA) was performed to identify informative principal components, and the top 30 principal components were selected for subsequent clustering and dimensionality reduction based on the ElbowPlot.

Clustering and Cell Type Annotation: In PCA space, a K-nearest neighbor graph was constructed using the FindNeighbors function based on Euclidean distance with default parameters and 30 principal components. The FindClusters function was used for clustering at a resolution of 0.6. Cell type annotation was performed using SingleR with the celldex package and refined according to marker gene references from the literature and public databases. Key marker genes included CD3D (T cells), CD79A (B cells), CD14 (monocytes), NCAM1 (natural killer cells), PECAM1 (endothelial cells), S100A9 (neutrophils), LYZ (myeloid cells), GATA1 (hematopoietic stem cells), and CD1C (dendritic cells). Dimensionality reduction visualization and dataset exploration were performed using the RunUMAP function.

Differential Gene Expression Analysis: Key gene expression levels across different cell types were displayed using the DotPlot and FeaturePlot functions. Differential expression between the AF and normal groups within each annotated cell type was analyzed using the Wilcoxon rank-sum test. Representative differentially expressed genes across cell types and groups were displayed using heatmaps.

### Statistical analysis

All data processing and analyses were conducted using R software (version 4.2.2). Continuous variables were tested for normality before statistical comparison. For comparisons between two groups, Student's *t*-test was used for normally distributed data, whereas the Mann–Whitney *U*-test (Wilcoxon rank-sum test) was used for non-normally distributed data. For comparisons involving three or more groups, one-way analysis of variance (ANOVA) or the Kruskal–Wallis test was used as appropriate. Correlation analyses between different molecules were conducted using Spearman's correlation analysis. Differences in immune cell infiltration and correlations between immune cells and key genes were analyzed with FDR correction using the Benjamini–Hochberg (BH) method, with FDR < 0.05 considered statistically significant. Unless otherwise noted, all statistical tests were two-sided, and *n* refers to biologically independent animals or independently repeated cell experiments rather than technical replicates.

## Results

### AMI model validation revealed increased arrhythmia burden and PVN inflammatory activation

**Figure 1:**
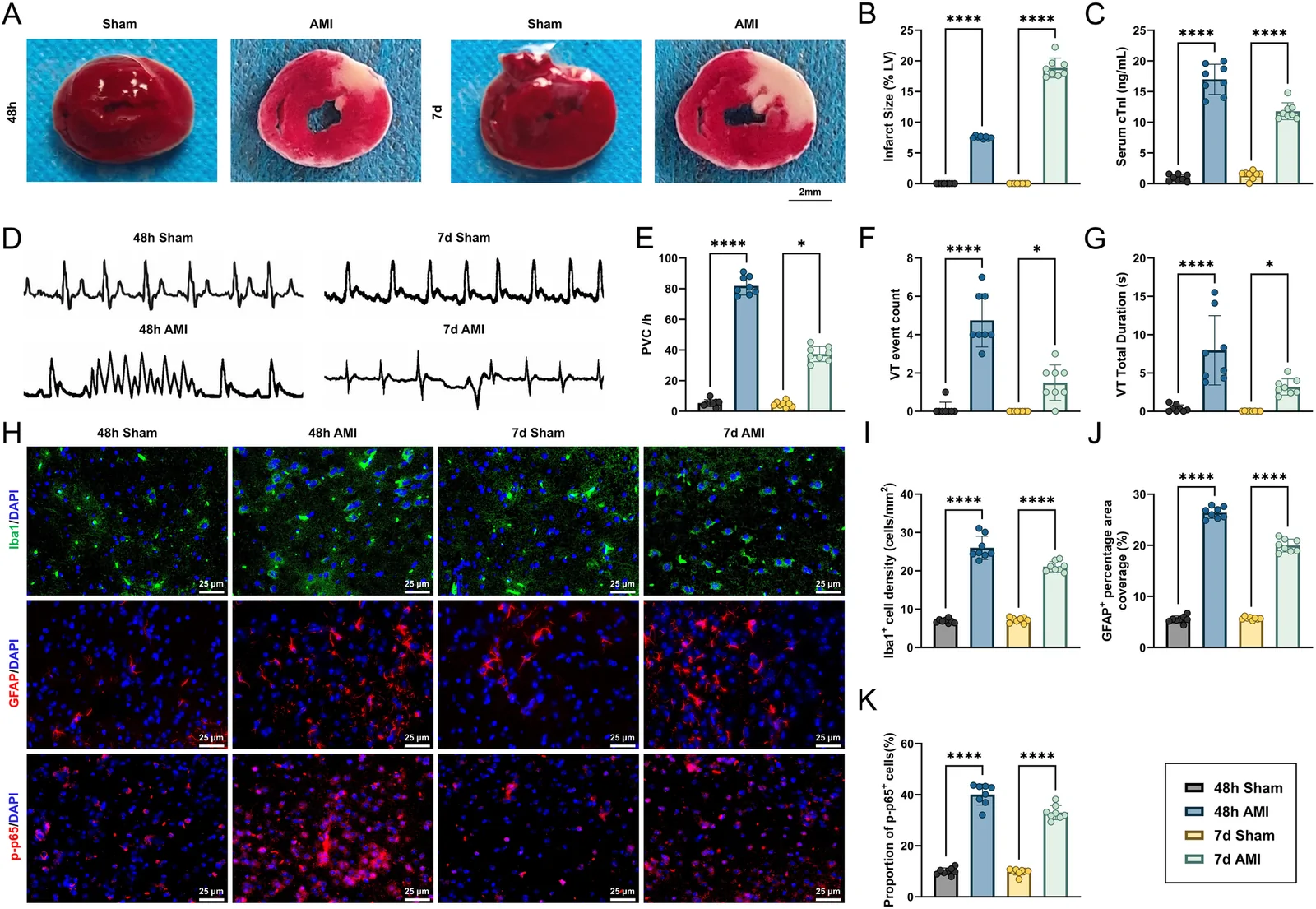
Myocardial infarction injury, arrhythmic phenotypes, and PVN inflammation/NF-*κ*B activation jointly confirm successful establishment of the AMI model. **(A)** TTC staining of left ventricular transverse sections from Sham and AMI groups at 48 h and 7 days after surgery. Red indicates viable myocardium, whereas white/light areas indicate infarcted regions; scale bar = 2 mm. **(B)** Quantitative analysis of TTC staining showing the proportion of left ventricular infarct area (% LV). **(C)** Serum cTnI levels measured by ELISA. **(D)** Representative ECG telemetry waveforms. **(E)** PVC burden (PVC/hour). **(F)** Number of VT episodes. **(G)** VA burden quantified by ECG telemetry as total VT duration (seconds). **(H)** Representative immunofluorescence images of the PVN showing Iba1 (microglial marker), GFAP (astrocytic marker), and p-p65 (marker of NF-*κ*B activation). **(I–K)** Quantitative analyses of PVN immunofluorescence: density of Iba1-positive cells (cells/mm^2^) **(I)**, percentage of GFAP-positive area (%) **(J)**, and proportion of p-p65-positive cells (%) **(K)**. Data are presented as box plots; *n* = 8 per group. *p < 0.05; ****p < 0.0001.

biochemical markers were assessed at 48 h and 7 d after surgery. TTC staining showed clear infarct regions in the AMI group at both time points, whereas the Sham group exhibited uniform staining without evident necrosis (Figure 1A). Quantitative analysis demonstrated that infarct size was significantly increased in the AMI group at both 48 h and 7 d compared with the corresponding Sham group (both comparisons, *p* < 0.0001) (Figure 1B), and the infarct area at 7 d was larger than that at 48 h. Serum cTnI levels were also significantly elevated in the AMI group at both time points compared with the corresponding Sham group (both comparisons, *p* < 0.0001), reaching a peak at 48 h and remaining higher than those in the Sham group at 7 d (Figure 1C). In contrast, serum cTnI levels remained low in the Sham group.

Representative ECG telemetry traces showed regular rhythms in the Sham group, whereas nonsustained VT and VT were observed at 48 h after AMI, and abnormal beats including PVCs were still evident at 7 d (Figure 1D). Quantitative analysis further showed that the AMI group exhibited a significantly increased PVC burden (PVCs/h) at both 48 h and 7 d compared with the corresponding Sham group (*p* < 0.0001 and *p* < 0.05, respectively) (Figure 1E). In addition, VT episode count was significantly increased at both 48 h and 7 d (*p* < 0.0001 and *p* < 0.05, respectively) (Figure 1F), and total VT duration was also markedly prolonged at both time points (*p* < 0.0001 and *p* < 0.05, respectively) (Figure 1G). These changes were more pronounced at 48 h, although they remained statistically significant at 7 d.

Immunofluorescence analysis of the PVN showed increased Iba1-positive microglia, enhanced GFAP signals, and elevated p-p65-positive signals in the AMI group relative to the Sham group at both time points (Figure 1H). Quantitative analysis confirmed increased Iba1-positive cell density (*p* < 0.0001) (Figure 1I), expanded GFAP-positive area coverage (*p* < 0.0001) (Figure 1J), and a higher proportion of p-p65-positive cells (*p* < 0.0001) (Figure 1K) in the AMI group. In summary, TTC staining and elevated serum cTnI confirmed successful establishment of the AMI model. AMI was accompanied by increased arrhythmia burden together with PVN glial activation and enhanced NF-*κ*B signaling, indicating a parallel association rather than direct proof of upstream causal control.

### Identification of DEGs and DEMs in the AMI dataset

**Figure 2:**
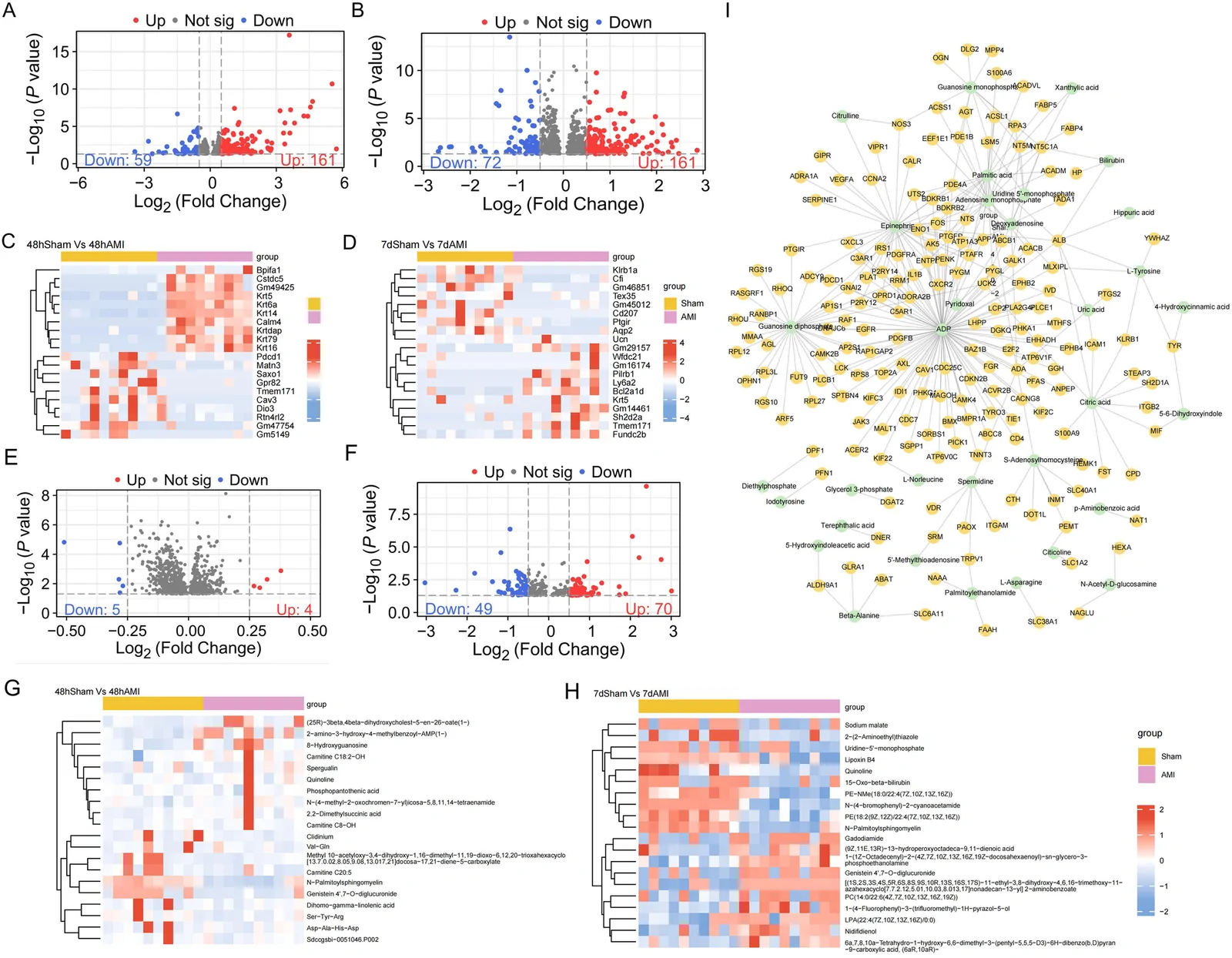
Differential expression analysis of genes and metabolites. **(A,B)** Volcano plots showing differential gene expression between the AMI and Sham groups in the transcriptomic data at 48 h and 7 days, respectively. **(C,D)** Heatmaps of the top 20 DEGs in the transcriptomic data at 48 h and 7 days; yellow indicates the Sham group and pink indicates the AMI group. In the heatmaps, red represents high expression and blue represents low expression. **(E,F)** Volcano plots of DEMs between the AMI and Sham groups in the metabolomic data at 48 h and 7 days. **(G,H)** Heatmaps of the top 20 DEMs at 48 h and 7 days in the AMI dataset; yellow indicates the Sham group and blue indicates the AMI group. In the heatmaps, red denotes high expression and blue denotes low expression. **(I)** Interaction network of DEGs and DEMs, in which yellow nodes represent DEGs and green nodes represent DEMs. AMI, Acute Myocardial Infarction.

To investigate the molecular changes in brain tissue induced by AMI, we first identified DEGs in the AMI transcriptomic dataset by comparing the 48-hour AMI group with the 48-hour sham group, and the 7-day AMI group with the 7-day sham group. In the 48-hour AMI group, a total of 220 DEGs met the screening criteria (|log_2_FC| ≥ 0.5, *p* < 0.05), including 161 upregulated and 59 downregulated genes (Supplementary Table S1). In the 7-day AMI group, 233 DEGs met the same criteria, including 161 upregulated and 72 downregulated genes (Supplementary Table S2). After merging and removing duplicates, a total of 412 DEGs were obtained (Supplementary Table S3). Volcano plots show the differential expression analysis results at 48 h (Figure 2A) and 7 days (Figure 2B), while heatmaps display the expression patterns of the top 20 DEGs ranked by |log2FC| (Figures 2C,D).

Next, in the AMI metabolomic dataset, we identified DEMs by comparing the 48-hour AMI group with the sham group, as well as the 7-day AMI group with the sham group. In the 48-hour AMI group, 9 DEMs met the screening criteria (|log_2_FC| ≥ 0.25, *p* < 0.05), including 4 upregulated and 5 downregulated metabolites (Supplementary Table S4). In the 7-day AMI group, 119 DEMs met the screening criteria (|log_2_FC| ≥ 0.5, *p* < 0.05), including 70 upregulated and 49 downregulated metabolites (Supplementary Table S5). After merging and removing duplicates, a total of 129 DEMs were obtained (Supplementary Table S6). Volcano plots illustrate the differential metabolite analysis results at 48 h (Figure 2E) and 7 days (Figure 2F), while heatmaps show the expression patterns of the top 20 DEMs ranked by |log_2_FC| (Figures 2G,H).

Gene–metabolite interaction analysis identified a network comprising 180 DEGs and 34 DEMs (Figure 2I; Supplementary Table S7), indicating a coordinated association between transcriptomic and metabolomic alterations in the AMI dataset.

### Identification of VA-associated candidate DEGs and DEMs by MR analysis

**Figure 3:**
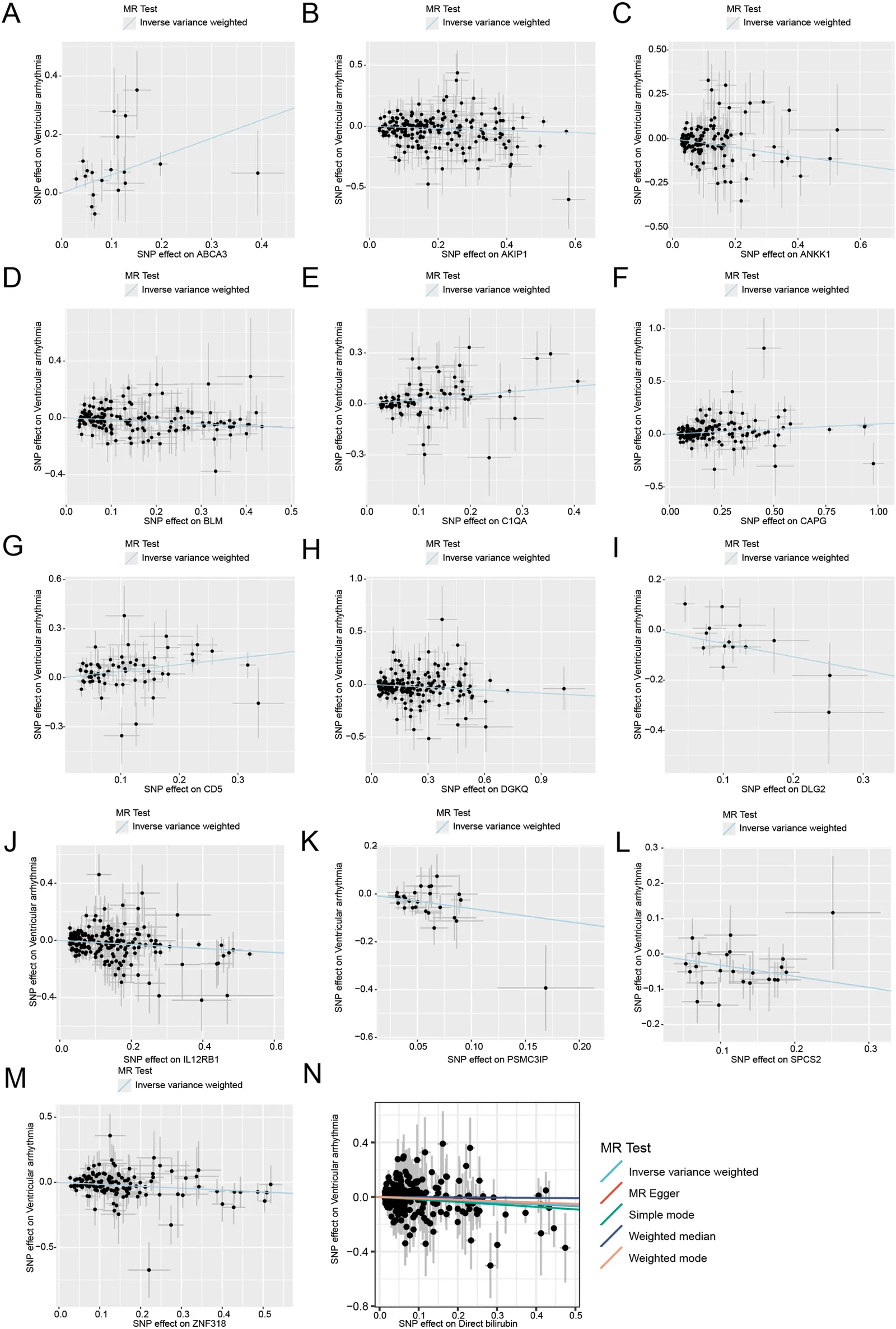
Scatter plot of causal effect estimates. **(A–M)** Scatter plots of MR effect estimates for ABCA3 **(A)**, AKIP1 **(B)**, ANKK1 **(C)**, BLM **(D)**, C1QA **(E)**, CAPG **(F)**, CD5 **(G)**, DGKQ **(H)**, DLG2 **(I)**, IL12RB1 **(J)**, PSMC3IP **(K)**, SPCS2 **(L)**, and ZNF318 **(M)** on VA. **(N)** Scatter plot of MR effect estimates for DEMs on VA. The slope of the fitted line represents the predicted strength of the causal relationship.

Differentially expressed genes identified through interspecies mapping and intersection with the eQTLGen database were selected for subsequent analysis (Supplementary Table S8). Using the predefined significance threshold, two-sample MR analysis identified 13 genes associated with VA risk (Table 2). Among these, ABCA3, CD5, C1QA and CAPG showed positive associations with VA risk, whereas AKIP1, DGKQ, BLM, IL12RB1, ZNF318, ANKK1, SPCS2, DLG2 and PSMC3IP showed negative associations (Figures 3A–M). For genes with three or more instrumental single-nucleotide polymorphisms, additional sensitivity analyses, including heterogeneity, horizontal pleiotropy, leave-one-out and Steiger directionality tests, did not indicate substantial bias (Tables 3–5 and Supplementary Table S9).

**Table 2:** Estimate of MR causal effect of DEGs on VA incidence.

**outcome**	**exposure**	**method**	**nsnp**	**Beta.**	**se**	**pvalue**	**OR (95% CI)**
Ventricular arrhythmia	CAPG	Inverse variance weighted	147	0.096	0.019	2.42E-07	1.10 (1.06, 1.14)
Ventricular arrhythmia	IL12RB1	Inverse variance weighted	203	-0.141	0.023	8.54E-10	0.87 (0.83, 0.91)
Ventricular arrhythmia	CD5	Inverse variance weighted	63	0.402	0.079	3.46E-07	1.50 (1.28, 1.75)
Ventricular arrhythmia	SPCS2	Inverse variance weighted	23	−0.316	0.073	1.56E-05	0.73 (0.63, 0.84)
Ventricular arrhythmia	PSMC3IP	Inverse variance weighted	27	−0.611	0.146	2.69E-05	0.54 (0.41, 0.72)
Ventricular arrhythmia	DGKQ	Inverse variance weighted	185	−0.092	0.017	3.96E-08	0.91 (0.88, 0.94)
Ventricular arrhythmia	DLG2	Inverse variance weighted	14	−0.534	0.124	1.66E-05	0.59 (0.46, 0.75)
Ventricular arrhythmia	AKIP1	Inverse variance weighted	195	−0.088	0.021	3.46E-05	0.92 (0.88, 0.95)
Ventricular arrhythmia	ABCA3	Inverse variance weighted	19	0.624	0.150	3.26E-05	1.87 (1.39, 2.51)
Ventricular arrhythmia	ANKK1	Inverse variance weighted	124	−0.250	0.048	1.74E-07	0.78 (0.71, 0.86)
Ventricular arrhythmia	ZNF318	Inverse variance weighted	136	−0.148	0.032	4.56E-06	0.86 (0.81, 0.92)
Ventricular arrhythmia	C1QA	Inverse variance weighted	85	0.259	0.056	3.90E-06	1.30 (1.16, 1.45)
Ventricular arrhythmia	BLM	Inverse variance weighted	149	−0.140	0.027	1.62E-07	0.87 (0.82, 0.92)

SNP, single nucleotide polymorphism; OR, odds ratio; CI, confidence interval; SE, Standard Error.

**Table 3:** Heterogeneity test of genes for VA by MR analysis.

**outcome**	**exposure**	**method**	**Q**	**Q_df**	**Q_pval**	***I*^2^(%)**
Ventricular arrhythmia	CAPG	MR Egger	117.7925	145	9.53E-01	0
Ventricular arrhythmia	CAPG	Inverse variance weighted	119.403	146	9.48E-01	0
Ventricular arrhythmia	IL12RB1	MR Egger	227.9283	201	9.34E-02	11.81
Ventricular arrhythmia	IL12RB1	Inverse variance weighted	230.4999	202	8.24E-02	12.36
Ventricular arrhythmia	CD5	MR Egger	68.03837	61	2.50E-01	10.34
Ventricular arrhythmia	CD5	Inverse variance weighted	68.15072	62	2.76E-01	9.03
Ventricular arrhythmia	SPCS2	MR Egger	15.05322	21	8.20E-01	0
Ventricular arrhythmia	SPCS2	Inverse variance weighted	17.16293	22	7.54E-01	0
Ventricular arrhythmia	PSMC3IP	MR Egger	20.40108	25	7.25E-01	0
Ventricular arrhythmia	PSMC3IP	Inverse variance weighted	20.40287	26	7.72E-01	0
Ventricular arrhythmia	DGKQ	MR Egger	175.379	183	6.44E-01	0
Ventricular arrhythmia	DGKQ	Inverse variance weighted	178.7672	184	5.95E-01	0
Ventricular arrhythmia	DLG2	MR Egger	13.85262	12	3.10E-01	13.37
Ventricular arrhythmia	DLG2	Inverse variance weighted	14.27878	13	3.55E-01	8.96
Ventricular arrhythmia	AKIP1	MR Egger	220.3436	193	8.62E-02	12.41
Ventricular arrhythmia	AKIP1	Inverse variance weighted	223.5405	194	7.17E-02	13.21
Ventricular arrhythmia	ABCA3	MR Egger	23.59204	17	1.31E-01	27.94
Ventricular arrhythmia	ABCA3	Inverse variance weighted	26.34398	18	9.21E-02	31.67
Ventricular arrhythmia	ANKK1	MR Egger	112.1814	122	7.27E-01	0
Ventricular arrhythmia	ANKK1	Inverse variance weighted	112.1821	123	7.48E-01	0
Ventricular arrhythmia	ZNF318	MR Egger	112.103	134	9.16E-01	0
Ventricular arrhythmia	ZNF318	Inverse variance weighted	112.9954	135	9.16E-01	0
Ventricular arrhythmia	C1QA	MR Egger	63.8979	83	9.41E-01	0
Ventricular arrhythmia	C1QA	Inverse variance weighted	66.39995	84	9.21E-01	0
Ventricular arrhythmia	BLM	MR Egger	108.401	147	9.93E-01	0
Ventricular arrhythmia	BLM	Inverse variance weighted	108.4198	148	9.94E-01	0

Q, Cochran Q-test statistic; Q_df, Q-test degree of freedom; Q_pval, *P* value of Q-test; The *I*^2^ statistic reflects the proportion of the heterogeneity part of the instrumental variable in the total variation: if *I*^2^ ≤ 0, set it to 0, indicating that no heterogeneity was observed; *I*^2^ = 0∼25%, indicating mild heterogeneity; *I*^2^ = 25% to 50%, indicating moderate heterogeneity; *I*^2^ > 50%, indicating high heterogeneity. Specific calculation formula: *I*^2^ = (Q-df)/Q × 100%.

**Table 4:** Horizontal pleiotropy test of MR analysis of genes for VA.

**outcome**	**exposure**	**egger_intercept**	**se**	**pvalue**
Ventricular arrhythmia	CAPG	7.65E-03	6.03E-03	2.06E-01
Ventricular arrhythmia	IL12RB1	8.01E-03	5.32E-03	1.34E-01
Ventricular arrhythmia	CD5	4.09E-03	1.29E-02	7.52E-01
Ventricular arrhythmia	PSMC3IP	-8.79E-04	2.08E-02	9.67E-01
Ventricular arrhythmia	DGKQ	-1.19E-02	6.46E-03	6.73E-02
Ventricular arrhythmia	SPCS2	1.71E-02	8.58E-03	4.92E-02
Ventricular arrhythmia	DLG2	2.93E-02	4.82E-02	5.55E-01
Ventricular arrhythmia	AKIP1	−1.08E-02	6.46E-03	9.59E-02
Ventricular arrhythmia	ABCA3	3.42E-02	2.43E-02	1.77E-01
Ventricular arrhythmia	ANKK1	1.86E-04	7.03E-03	9.79E-01
Ventricular arrhythmia	ZNF318	6.86E-03	7.26E-03	3.47E-01
Ventricular arrhythmia	C1QA	−1.31E-02	8.25E-03	1.18E-01
Ventricular arrhythmia	BLM	8.74E-04	6.37E-03	8.91E-01

SE, Standard Error.

**Table 5:** Steiger directivity test for MR analysis of genes for VA.

**exposure**	**outcome**	**snp_r2.exposure**	**snp_r2.outcome**	**correct_causal_direction**	**steiger_pval**
CAPG	Ventricular arrhythmia	1.291846713	0.000444999	TRUE	
IL12RB1	Ventricular arrhythmia	0.939330665	0.000833277	TRUE	0
CD5	Ventricular arrhythmia	0.118073005	0.000294709	TRUE	0
SPCS2	Ventricular arrhythmia	0.07716996	0.000109073	TRUE	3.62E-136
PSMC3IP	Ventricular arrhythmia	0.020703644	0.000115833	TRUE	1.17E-105
DGKQ	Ventricular arrhythmia	1.318794397	0.000636627	TRUE	
DLG2	Ventricular arrhythmia	0.032723493	0.00010555	TRUE	1.09E-66
AKIP1	Ventricular arrhythmia	1.045018051	0.00,07,41199	TRUE	
ABCA3	Ventricular arrhythmia	0.03,54,14056	0.00,01,57247	TRUE	1.94E-155
ANKK1	Ventricular arrhythmia	0.26,36,21688	0.00042506	TRUE	0
ZNF318	Ventricular arrhythmia	0.520527839	0.000408265	TRUE	0
C1QA	Ventricular arrhythmia	0.193045486	0.000267247	TRUE	0
BLM	Ventricular arrhythmia	0.621509944	0.000414002	TRUE	0

SNP, single nucleotide polymorphism; *r*^2^, variance explanation rate.

Among the screened DEMs, 153 metabolite-related GWAS traits were retained as exposure variables (Supplementary Table S10). Two-sample MR analysis indicated that direct bilirubin was negatively associated with VA risk (Table 6). The effect estimates were directionally consistent across the five MR models, and no significant heterogeneity, horizontal pleiotropy or directional inconsistency was detected (Figure 3N; Tables 7–9 and Supplementary Table S11).

**Table 6:** MR causal effect estimation of DEMs on VA pathogenesis.

**outcome**	**exposure**	**method**	**nsnp**	***β***	**se**	**pvalue**	**OR (95% CI)**
Ventricular arrhythmia	Direct bilirubin	MR Egger	299	−0.136	0.054	1.20E-02	0.873 (0.786,0.970)
Ventricular arrhythmia	Direct bilirubin	Weighted median	299	-0.017	0.070	8.03E-01	0.983 (0.857,1.126)
Ventricular arrhythmia	Direct bilirubin	Inverse variance weighted	299	-0.131	0.041	1.55E-03	0.87 (0.809,0.951)
Ventricular arrhythmia	Direct bilirubin	Simple mode	299	-0.181	0.112	1.07E-01	0.834 (0.669,1.040)
Ventricular arrhythmia	Direct bilirubin	Weighted mode	299	-0.101	0.058	8.48E-02	0.904 (0.806,1.014)

SNP, single nucleotide polymorphism; OR, odds ratio; CI, confidence interval; SE, Standard Error.

**Table 7:** Heterogeneity test of DEMs for VA by MR analysis.

**outcome**	**exposure**	**method**	**Q**	**Q_df**	**Q_pval**	***I*^2^(%)**
Ventricular arrhythmia	Direct bilirubin	MR Egger	284.2845	297	0.692132	0
Ventricular arrhythmia	Direct bilirubin	Inverse variance weighted	284.3064	298	0.706202	0

: Q, Cochran Q-test statistic; Q_df, Q-test degree of freedom; Q_pval, *P* value of Q-test; The *I*^2^ statistic reflects the proportion of the heterogeneity part of the instrumental variable in the total variation: if *I*^2^ ≤ 0, set it to 0, indicating that no heterogeneity was observed; *I*^2^ = 0∼25%, indicating mild heterogeneity; *I*^2^ = 25% to 50%, indicating moderate heterogeneity; *I*^2^ > 50%, indicating high heterogeneity. Specific calculation formula: *I*^2^ = (Q-df)/Q × 100%.

**Table 8:** Level pleiotropy test of MR analysis for DEMs on VA.

**outcome**	**exposure**	**egger_intercept**	**se**	***P* value**
Ventricular arrhythmia	Direct bilirubin	5.38E-04	3.64E-03	8.82E-01

SE, Standard Error.

**Table 9:** MR analysis of DEMs for VA steiger directivity test.

**exposure**	**outcome**	**snp_r2.exposure**	**snp_r2.outcome**	**correct_causal_direction**	**steiger_pval**
Direct bilirubin	Ventricular arrhythmia	2.11E-01	8.97E-04	TRUE	0

SNP, single nucleotide polymorphism; *r*^2^, variance explanation rate.

SMR analysis further prioritized five genes—DGKQ, BLM, ANKK1, ABCA3 and IL12RB1—as significantly associated with VA (*p*_SMR < 0.05, Table 10). For these genes, *p*_HEIDI values were all > 0.05, suggesting that the observed associations were less likely to be explained by linkage. Because the eQTL instruments were blood-derived, these results should be interpreted as evidence supporting genetically predicted expression associated with VA risk rather than tissue-specific effects in the PVN.

**Table 10:** SMR analysis results of genes for VA.

exposure	outcome	topSNP	Beta.	p_SMR	p_HEIDI
C1QA	ebi-a-GCST90018940	rs12058824	0.33	7.09E-02	7.18E-01
CAPG	ebi-a-GCST90018940	rs35666320	0.06	3.47E-01	7.65E-01
DGKQ	ebi-a-GCST90018940	rs62294486	-0.08	4.12E-02	3.74E-01
ZNF318	ebi-a-GCST90018940	rs70953670	-0.15	2.62E-01	7.53E-01
AKIP1	ebi-a-GCST90018940	rs10840147	−0.07	1.24E-01	2.23E-01
CD5	ebi-a-GCST90018940	rs3019567	0.25	3.31E-01	2.71E-01
SPCS2	ebi-a-GCST90018940	rs1315299	−0.21	2.43E-01	6.70E-01
DLG2	ebi-a-GCST90018940	rs616320	−0.44	9.22E-02	/
ANKK1	ebi-a-GCST90018940	rs6589371	−0.30	2.53E-02	6.00E-01
BLM	ebi-a-GCST90018940	rs378996	−0.17	3.26E-02	9.55E-01
ABCA3	ebi-a-GCST90018940	rs17135889	0.50	1.70E-02	9.75E-01
PSMC3IP	ebi-a-GCST90018940	rs12938909	−0.28	3.80E-01	5.31E-01
IL12RB1	ebi-a-GCST90018940	rs17885551	−0.18	3.65E-03	6.54E-01

SNP, single nucleotide polymorphism; SMR, Summary-data-based Mendelian Randomization; HEIDI, Heterogeneity in Dependent Instruments.

### *In vivo* bilirubin intervention reduced post-AMI arrhythmia burden and suppressed PVN inflammation/NF-*κ*B activation

**Figure 4:**
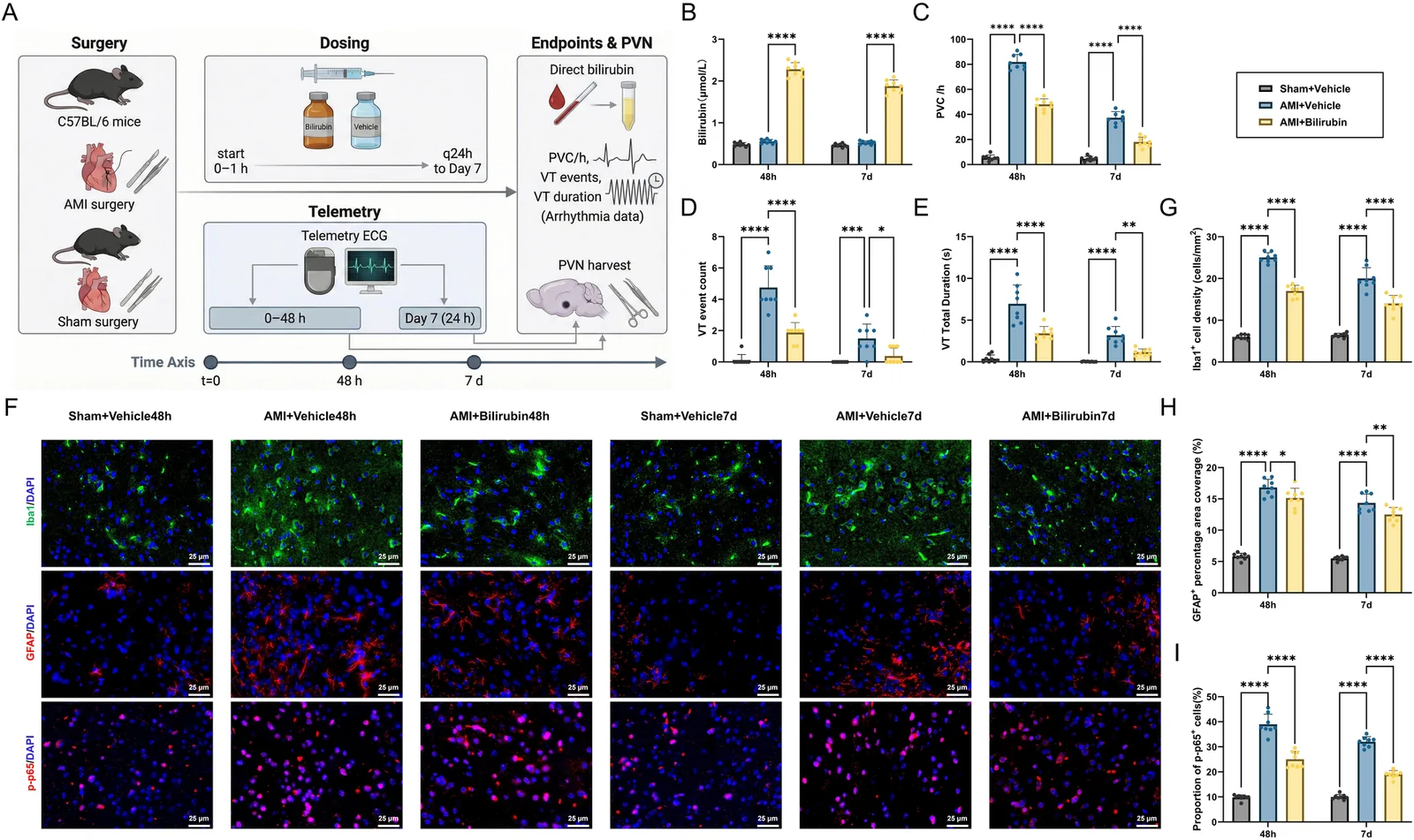
*In vivo* bilirubin intervention increased Serum direct bilirubin levels and reduced post-AMI arrhythmia burden while suppressing PVN inflammation and NF-*κ*B activation. **(A)** Schematic of the experimental workflow: C57BL/6 mice underwent AMI or Sham surgery, followed by administration of bilirubin or vehicle starting 0-1 h after surgery (once every 24 h until day 7). ECG telemetry was recorded continuously during the 0-48 h postoperative period and for an additional 24 h on day 7. Blood samples were collected at 48 h and 7 days to measure direct bilirubin levels, and PVN tissues were harvested for immunofluorescence analysis. **(B)** Serum direct bilirubin levels at 48 h and 7 days. **(C)** PVC burden (PVC/hour). **(D)** Number of VT episodes. **(E)** Total VT duration (seconds). **(F)** Representative immunofluorescence images of the PVN showing Iba1 (microglia, green), GFAP (astrocytes, red), p-p65 (NF-*κ*B activation, red), and DAPI (nuclei, blue). **(G)** Density of Iba1⁺ cells in the PVN (cells/mm^2^). **(H)** Percentage of GFAP⁺ area coverage in the PVN. **(I)** Proportion of p-p65⁺ cells in the PVN (%).

*In vivo bilirubin intervention* was evaluated in the mouse AMI model at 48 h and 7 d, with assessment of serum direct bilirubin, ventricular arrhythmia burden, and PVN inflammatory markers (Figure 4A). Compared with the corresponding vehicle-treated groups, bilirubin intervention significantly increased serum direct bilirubin levels at both time points (both comparisons, *p* < 0.0001) (Figure 4B).

Relative to the AMI + Vehicle group, bilirubin intervention reduced post-AMI PVC burden at both 48 h and 7 d (both comparisons, *p* < 0.0001) (Figure 4C). Bilirubin treatment also decreased VT episode count at 48 h and 7 d (*p* < 0.0001 and *p* < 0.05, respectively) (Figure 4D), and shortened total VT duration at both time points (*p* < 0.0001 at 48 h and *p* < 0.01 at 7 d) (Figure 4E). These effects were more pronounced at 48 h but remained evident at 7 d.

Immunofluorescence analysis of the PVN showed increased Iba1, GFAP, and p-p65 signals in the AMI + Vehicle group at both 48 h and 7 d, whereas these signals were reduced in the AMI + Bilirubin group (Figure 4F). Quantitative analysis confirmed that bilirubin treatment reduced Iba1-positive cell density at both time points (both comparisons, *p* < 0.0001) (Figure 4G), decreased GFAP-positive area coverage at 48 h and 7 d (*p* < 0.05 and *p* < 0.01, respectively) (Figure 4H), and lowered the proportion of p-p65-positive cells at both time points (both comparisons, *p* < 0.0001) (Figure 4I)*.* These findings suggest that bilirubin intervention was associated with reduced post-AMI arrhythmia burden together with attenuation of PVN glial activation and NF-*κ*B signaling.

### Bayesian colocalization analysis does not identify strong shared genetic signals for the prioritized genes in VA

**Figure 5:**
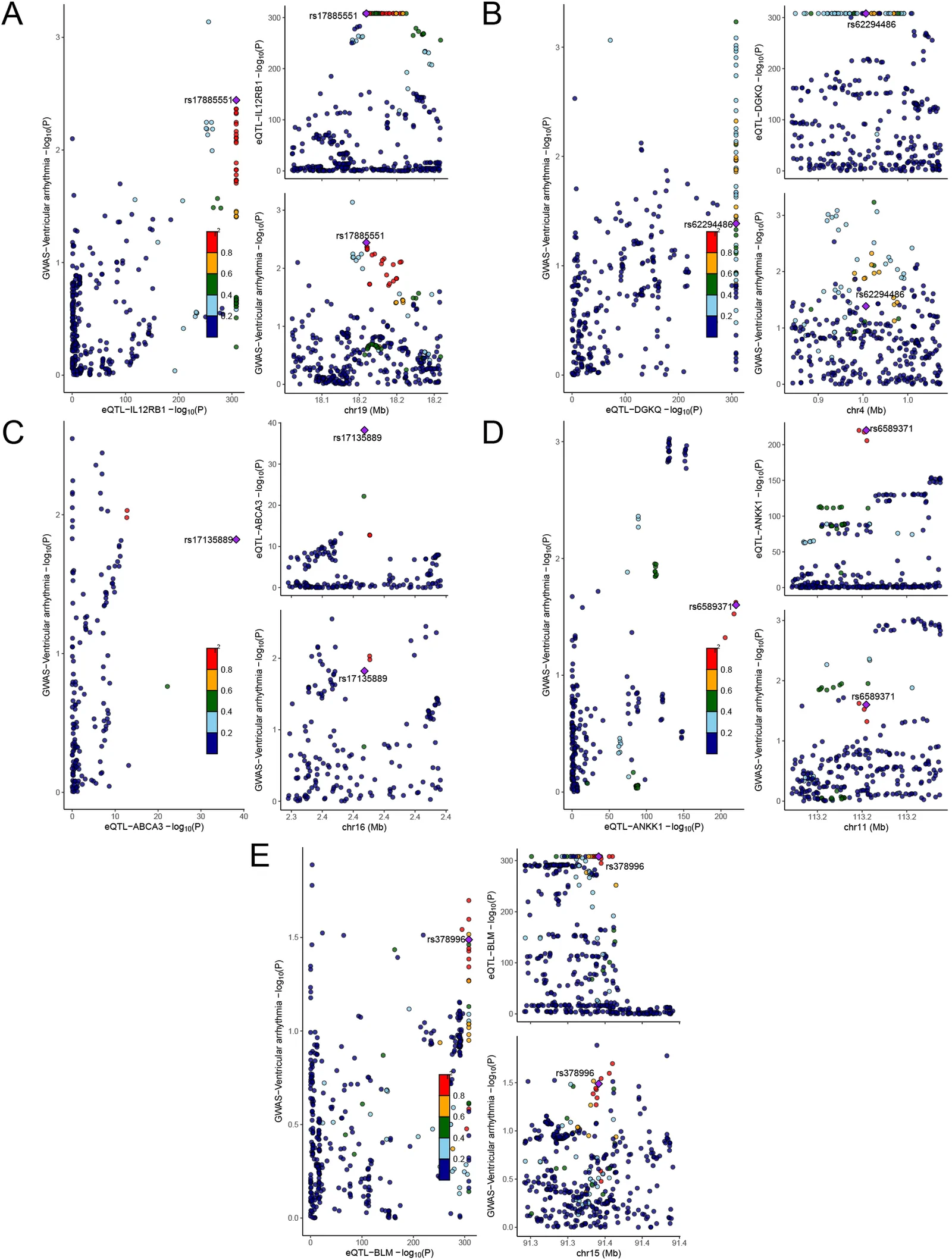
Co-Localization regions of genes and VA. **(A–E)** Co-localization regions of genes and VA: IL12RB1 **(A)**, DGKQ **(B)**, ABCA3 **(C)**, ANKK1 **(D)**, BLM **(E)** The left panel of each figure illustrates the distribution of SNPs' -log_10_**(*p*)** values from GWAS and eQTL studies, where smaller *p*-values correspond to higher positions on the *Y*-axis. The two right panels respectively show the distribution of SNP loci in eQTL and GWAS data, with the horizontal axis representing the SNP location and the vertical axis indicating the -log_10_**(*p*)** value of the SNP in GWAS or eQTL data.

Bayesian colocalization analysis was performed for the five genes prioritized in the previous steps to assess whether their association signals overlapped with those of VA at the same loci. Summary coloc results are shown in Table 11, and the detailed Bayesian colocalization results are provided in Supplementary Table S12. Across these genes, PP.H4 values ranged from 0.112 to 0.248, with ABCA3 showing the highest value (PP.H4 = 0.248), followed by IL12RB1 (PP.H4 = 0.220), DGKQ (PP.H4 = 0.210), ANKK1 (PP.H4 = 0.192), and BLM (PP.H4 = 0.112). None of the genes met the predefined threshold for moderate or strong colocalization. Locuscompare plots for IL12RB1, DGKQ, ABCA3, ANKK1, and BLM are shown in Figures 5A–E. Overall, the colocalization analysis did not provide strong evidence for shared causal variants between these candidate genes and VA at the tested loci.

**Table 11:** Results of coloc co-localization analysis of genes and arrhythmias.

**exposure**	**outcome**	**nsnps**	**PP.H0.abf**	**PP.H1.abf**	**PP.H2.abf**	**PP.H3.abf**	**PP.H4.abf**
DGKQ	Ventricular arrhythmia	334	0	0.743	0	0.048	0.210
ANKK1	Ventricular arrhythmia	267	2.1919E-213	0.740	2.0213E-214	0.068	0.192
BLM	Ventricular arrhythmia	431	0	0.866	0	0.022	0.112
ABCA3	Ventricular arrhythmia	164	1.67895E-32	0.735	3.83154E-34	0.017	0.248
IL12RB1	Ventricular arrhythmia	408	0	0.750	0	0.030	0.220

nsnps, Number of single nucleotide polymorphism.

### Validation of candidate genes in the AF cohort and molecular docking suggests potential compound interactions

**Figure 6:**
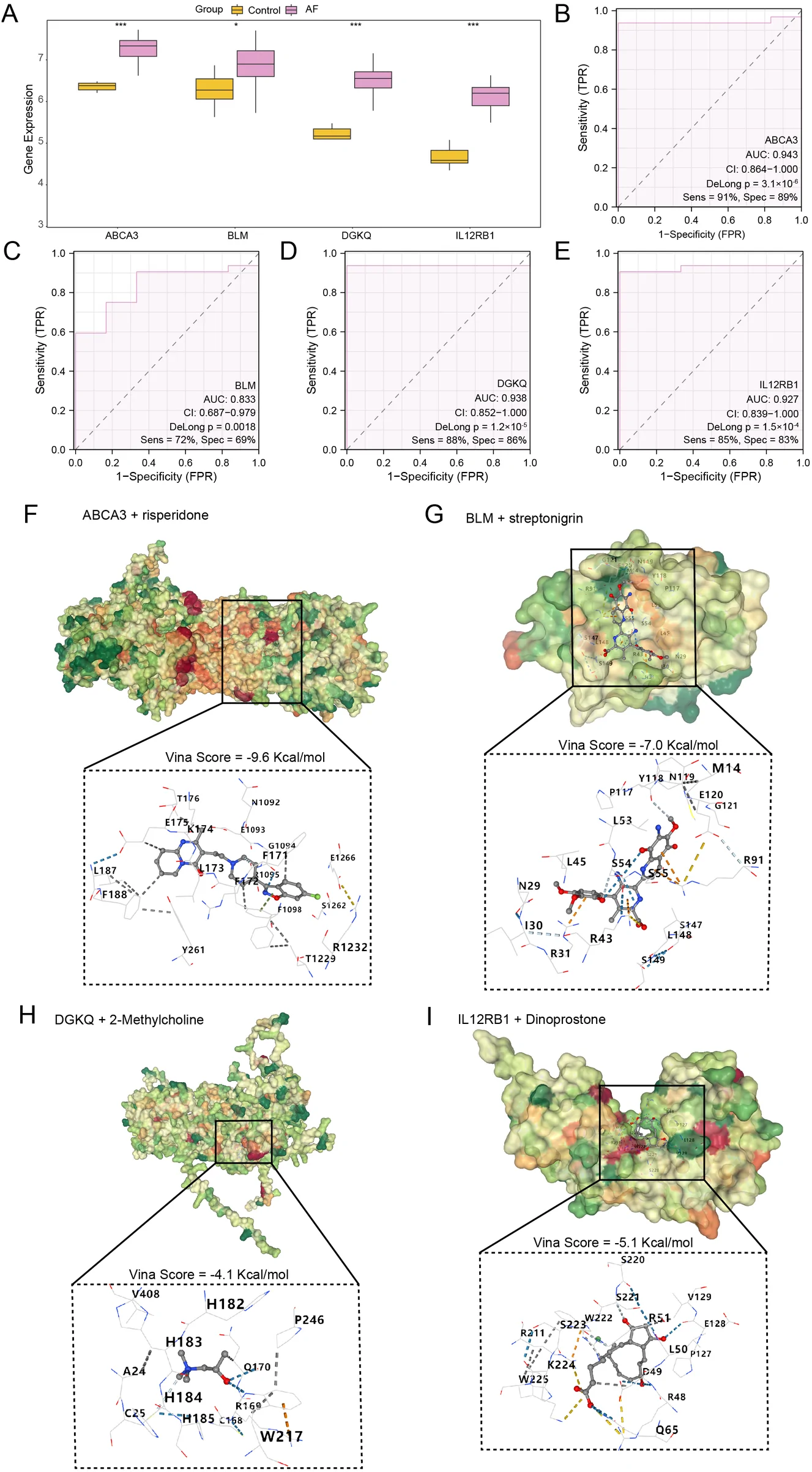
Diagnostic efficacy of genes and molecular docking analysis. **(A)** Box plots depicting the expression levels of key genes across different groups in GSE41177; **(B–E)** ROC curves for genes ABCA3 **(B)**, BLM **(C)**, DGKQ **(D)**, and IL12RB1 **(E)** in GSE41177; **(F–I)** Visualization of molecular docking results: ABCA3 with risperidone **(F)**, BLM with streptonigrin **(G)**, DGKQ with 2-methylcholine **(H)**, and IL12RB1 with dinoprostone **(I)**. Each panel presents an overall docking view followed by an interaction force diagram. The color gradient on the protein surface transitions from green to orange to red, indicating a progression from hydrophilic to hydrophobic amino acids. Blue dashed lines represent hydrogen bonds, light blue dashed lines denote weak hydrogen bonds, gray dashed lines indicate hydrophobic interactions, yellow dashed lines signify ionic bonds, green dashed lines represent π-π stacking interactions, and orange dashed lines denote cation-π interactions. AF, Atrial Fibrillation; ROC, Receiver Operating Characteristic; AUC, Area Under the Curve; TPR, True Positive Rate; FPR, False Positive Rate. Yellow indicates the control group, while pink represents the AF group. * indicates statistical significance (*p* < 0.05); *** denotes highly significant differences (*p* < 0.001).

The GSE41177 cohort was used as an external human atrial-tissue validation dataset rather than as direct evidence of PVN-specific biology. Differential expression analysis in this dataset showed that DGKQ, BLM, ABCA3 and IL12RB1 were significantly differentially expressed in the AF group relative to controls (*p* < 0.05), whereas ANKK1 was not detected (Figure 6A).

ROC analysis was used to assess the discriminatory performance of these genes within the GSE41177 cohort. The AUC was 0.833 for BLM (95% CI: 0.687–0.979; DeLong test *p* = 0.0018), 0.938 for DGKQ (95% CI: 0.852–1.000; DeLong test *p* = 1.2 × 10^−5^), 0.943 for ABCA3 (95% CI: 0.864–1.000; DeLong test *p* = 3.1 × 10^−6^), and 0.927 for IL12RB1 (95% CI: 0.839–1.000; DeLong test *p* = 1.5 × 10^−4^ (Figures 6B–E). The corresponding sensitivities were 72%, 88%, 91% and 85%, and the specificities were 69%, 86%, 89% and 83%, respectively.

Drug–gene association analysis identified candidate compounds linked to the four genes (Supplementary Table S13). Molecular docking further showed predicted interactions for ABCA3-risperidone, BLM-streptonigrin, DGKQ-2-Methylcholine and IL12RB1-dinoprostone, with Vina scores of −9.6, −7.0, −4.1 and −5.1 kcal/mol, respectively (Table 12; Figures 6F–I). Overall, the external AF atrial-tissue dataset supported differential expression of four candidate genes, and docking analysis suggested potential compound interactions for follow-up evaluation.

**Table 12:** TOP1 drug candidates for key genes.

**Term**	**P.value**	**Adjusted.P.value**	**Odds.Ratio**	**Combined.Score**	**Genes**
risperidone	9.57E-03	3.38E-02	141.4822695	657.8258626	ABCA3
streptonigrin	2.20E-03	3.11E-02	666.2	4,077.18792	BLM
2-Methylcholine	3.71E-02	6.63E-02	11.01682692	36.27975482	DGKQ
Dinoprostone	2.52E-02	4.91E-02	52.56613757	193.5731498	IL12RB1

### Correlation, chromosomal localization, and functional annotation of key genes in AF

**Figure 7:**
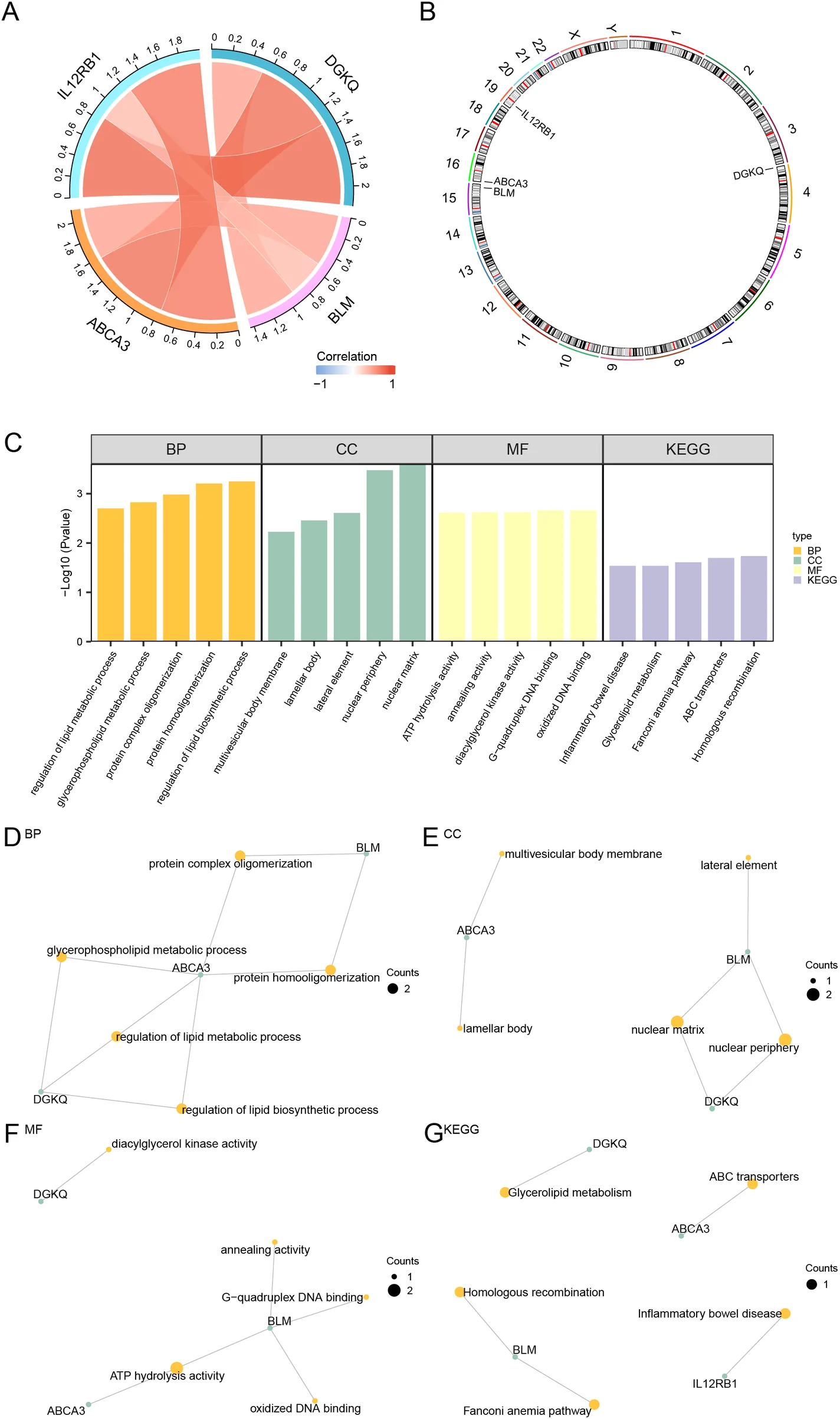
Correlation and functional annotation analyses of Key genes. **(A)** Correlation analysis of the four key genes in the GSE41177 dataset, visualized using a chord diagram; **(B)** Chromosomal localization of the key genes; **(C)** Bar plots showing GO and KEGG functional annotation results for the key genes, including BP, CC, MF, and biological pathways (KEGG); the *x*-axis represents GO and KEGG terms; **(D–G)** Network diagrams displaying GO and KEGG enrichment analysis results for key genes: BP **(D)**, CC **(E)**, MF **(F)**, KEGG **(G)** GO: Gene Ontology; KEGG: Kyoto Encyclopedia of Genes and Genomes; BP: Biological Process; CC: Cellular Component; MF: Molecular Function. The selection criteria for GO and KEGG enrichment analysis were *p*-value < 0.05 and FDR (*q*-value) < 0.25. In the network diagrams, orange nodes represent terms, green nodes represent molecules, and lines indicate relationships between terms and molecules.

Correlation analysis of the four key genes in the GSE41177 dataset showed broadly positive correlations among DGKQ, BLM, ABCA3 and IL12RB1 (Figure 7A). Chromosomal mapping showed that these genes were located on chromosomes 4, 15, 16 and 19 (Figure 7B).

Functional annotation analysis identified enrichment across BP, CC, MF, and KEGG categories (Figure 7C; Table 13). In BP, the enriched terms included regulation of lipid biosynthetic process, protein homooligomerization, protein complex oligomerization, glycerophospholipid metabolic process and regulation of lipid metabolic process. In CC, enriched terms included nuclear matrix, nuclear periphery, lateral element, lamellar body and multivesicular body membrane. In MF, enriched terms included oxidized DNA binding, G-quadruplex DNA binding, diacylglycerol kinase activity, annealing activity and ATP hydrolysis activity. In KEGG, the enriched pathways included homologous recombination, ABC transporters, Fanconi anemia pathway, glycerolipid metabolism and inflammatory bowel disease. Gene-term interaction networks are shown in Figures 7D–G. Overall, these results provide directional evidence that the key genes may participate in AF-related metabolic, structural and stress-response processes.

**Table 13:** Results of GO and KEGG enrichment analysis for key genes.

**ONTOLOGY**	**ID**	**Description**	**GeneRatio**	**BgRatio**	**pvalue**	**p.adjust**	**qvalue**
BP	GO:0046890	regulation of lipid biosynthetic process	2/4	182/18,614	5.63E-04	3.14E-02	7.66E-03
BP	GO:0051260	protein homooligomerization	2/4	191/18,614	6.20E-04	3.14E-02	7.66E-03
BP	GO:0051259	protein complex oligomerization	2/4	248/18,614	1.04E-03	3.14E-02	7.66E-03
BP	GO:0006650	glycerophospholipid metabolic process	2/4	297/18,614	1.49E-03	3.14E-02	7.66E-03
BP	GO:0019216	regulation of lipid metabolic process	2/4	344/18,614	1.99E-03	3.14E-02	7.66E-03
CC	GO:0016363	nuclear matrix	2/4	127/19,518	2.50E-04	4.18E-03	1.94E-03
CC	GO:0034399	nuclear periphery	2/4	147/19,518	3.35E-04	4.18E-03	1.94E-03
CC	GO:0000800	lateral element	1/4	12/19,518	2.46E-03	2.05E-02	9.48E-03
CC	GO:0042599	lamellar body	1/4	17/19,518	3.48E-03	2.17E-02	1.01E-02
CC	GO:0032585	multivesicular body membrane	1/4	29/19,518	5.93E-03	2.77E-02	1.28E-02
MF	GO:0032356	oxidized DNA binding	1/4	10/18,369	2.18E-03	1.36E-02	1.30E-03
MF	GO:0051880	G-quadruplex DNA binding	1/4	10/18,369	2.18E-03	1.36E-02	1.30E-03
MF	GO:0004143	diacylglycerol kinase activity	1/4	11/18369	2.39E-03	1.36E-02	1.30E-03
MF	GO:0140666	annealing activity	1/4	11/18,369	2.39E-03	1.36E-02	1.30E-03
MF	GO:0016887	ATP hydrolysis activity	2/4	376/18,369	2.44E-03	1.36E-02	1.30E-03
KEGG	hsa03440	Homologous recombination	1/4	41/8,850	1.84E-02	6.23E-02	1.51E-02
KEGG	hsa02010	ABC transporters	1/4	45/8,850	2.02E-02	6.23E-02	1.51E-02
KEGG	hsa03460	Fanconi anemia pathway	1/4	55/8,850	2.46E-02	6.23E-02	1.51E-02
KEGG	hsa00561	Glycerolipid metabolism	1/4	65/8,850	2.91E-02	6.23E-02	1.51E-02
KEGG	hsa05321	Inflammatory bowel disease	1/4	65/8,850	2.91E-02	6.23E-02	1.51E-02

GO, Gene Ontology; BP, Biological Process; CC, Cellular Component; MF, Molecular Function; KEGG, Kyoto Encyclopedia of Genes and Genomes.

### Differential expression analysis and GSEA reveal inflammation- and hypoxia-related pathways in AF

**Figure 8:**
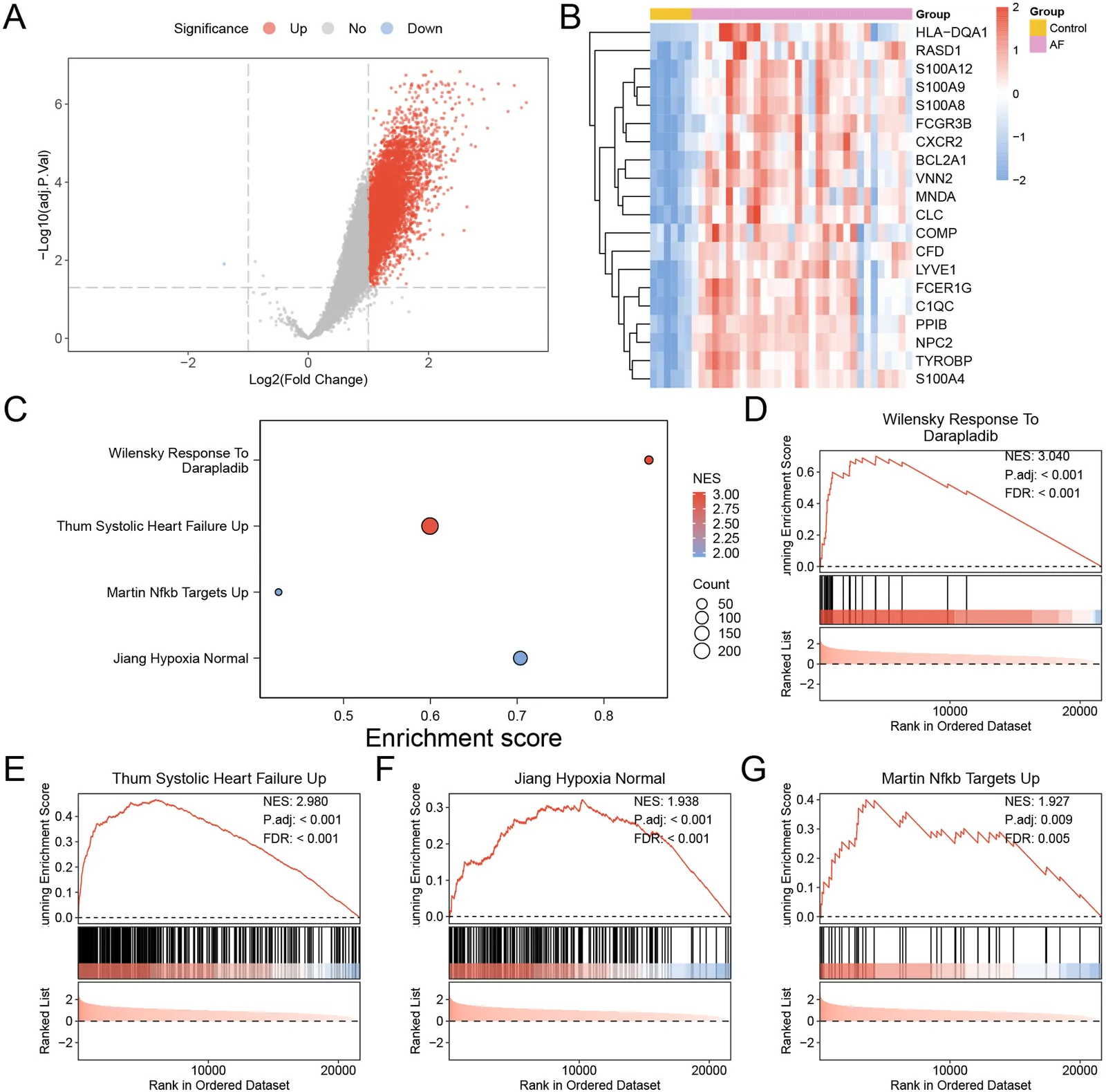
Differential gene expression and GSEA. **(A,B)** Volcano plot **(A)** and heatmap **(B)** of DEGs between the AF and control groups in GSE41177; **(C)** GSEA bubble plot for GSE41177; **(D–G)** GSEA results indicate upregulated pathways in the AF group, including Wilensky Dallapadula response, Thum contractile heart failure, Jiang normal hypoxia response, and Martin NF-*κ*B target genes. AF: atrial fibrillation. Pink represents the AF group, while yellow indicates the control group. In the bubble plot, the size of the bubbles corresponds to the size of the gene sets, with color denoting the normalized enrichment score (NES); redder colors indicate higher NES values, while bluer colors indicate lower NES values. In the heatmap, red indicates high expression, while blue denotes low expression. GSEA selection criteria were adj.*p* < 0.05 and FDR value (*q*-value) < 0.25, with *p*-values adjusted using the BH method.

Differential expression analysis was performed in the external AF atrial-tissue cohort GSE41177 to examine whether AF-associated cardiac transcriptional changes showed partial concordance with the inflammatory signatures suggested by the PVN-centered analyses. Using the predefined threshold of |log_2_FC| > 1 and adjusted *p* < 0.05, 9,225 DEGs were identified, including 9,224 upregulated genes and 1 downregulated gene (Figure 8A). The expression patterns of the top 20 DEGs ranked by absolute |log2FC| are shown in the heatmap (Figure 8B).

GSEA of the ranked gene list identified significant enrichment of multiple AF-associated gene sets (Figure 8C; Table 14). Among the highlighted pathways, WILENSKY_RESPONSE_TO_DARAPLIDIB (NES = 3.040, adjusted *p* < 0.001), THUM_SYSTOLIC_HEART_FAILURE_UP (NES = 2.980, adjusted *p* < 0.001), JIANG_HYPOXIA_NORMAL (NES = 1.938, adjusted *p* < 0.001), and MARTIN_NFKB_TARGETS_UP (NES = 1.927, adjusted *p* = 0.009) were significantly enriched in the AF group (Figures 8D–G; Table 14). Overall, the external AF atrial-tissue dataset showed enrichment of inflammation-, stress-, and hypoxia-related transcriptional programs.

**Table 14:** Results of GSEA analysis for combined datasets.

**ID**	**setSize**	**enrichmentScore**	**NES**	**pvalue**	**p.adjust**	**qvalue**
WILENSKY_RESPONSE_TO_DARAPLADIB	27	0.701	3.040	1.34E-10	5.23E-09	2.83E-09
THUM_SYSTOLIC_HEART_FAILURE_UP	397	0.467	2.980	1.00E-10	4.13E-09	2.23E-09
JIANG_HYPOXIA_NORMAL	189	0.322	1.938	8.98E-07	1.43E-05	7.73E-06
MARTIN_NFKB_TARGETS_UP	40	0.401	1.927	2.16E-03	8.98E-03	4.86E-03
MCLACHLAN_DENTAL_CARIES_UP	233	0.655	4.008	1.00E-10	4.13E-09	2.23E-09
FLECHNER_BIOPSY_KIDNEY_TRANSPLANT_REJECTED_VS_OK_UP	84	0.683	3.759	1.00E-10	4.13E-09	2.23E-09
NAKAYAMA_SOFT_TISSUE_TUMORS_PCA1_UP	75	0.690	3.720	1.00E-10	4.13E-09	2.23E-09
WIELAND_UP_BY_HBV_INFECTION	93	0.650	3.620	1.00E-10	4.13E-09	2.23E-09
NADLER_OBESITY_UP	57	0.688	3.535	1.00E-10	4.13E-09	2.23E-09
WP_TYROBP_CAUSAL_NETWORK_IN_MICROGLIA	59	0.663	3.428	1.00E-10	4.13E-09	2.23E-09
KHETCHOUMIAN_TRIM24_TARGETS_UP	45	0.685	3.385	1.00E-10	4.13E-09	2.23E-09
WALLACE_PROSTATE_CANCER_RACE_UP	272	0.545	3.372	1.00E-10	4.13E-09	2.23E-09
KIM_GLIS2_TARGETS_UP	81	0.611	3.342	1.00E-10	4.13E-09	2.23E-09
ICHIBA_GRAFT_VERSUS_HOST_DISEASE_35D_UP	132	0.574	3.339	1.00E-10	4.13E-09	2.23E-09
HESS_TARGETS_OF_HOXA9_AND_MEIS1_DN	81	0.603	3.296	1.00E-10	4.13E-09	2.23E-09
JAATINEN_HEMATOPOIETIC_STEM_CELL_DN	221	0.538	3.279	1.00E-10	4.13E-09	2.23E-09
MA_RAT_AGING_DN	147	0.558	3.277	1.00E-10	4.13E-09	2.23E-09

### Immune cell infiltration landscape and key gene associations suggest immune remodeling in AF

**Figure 9:**
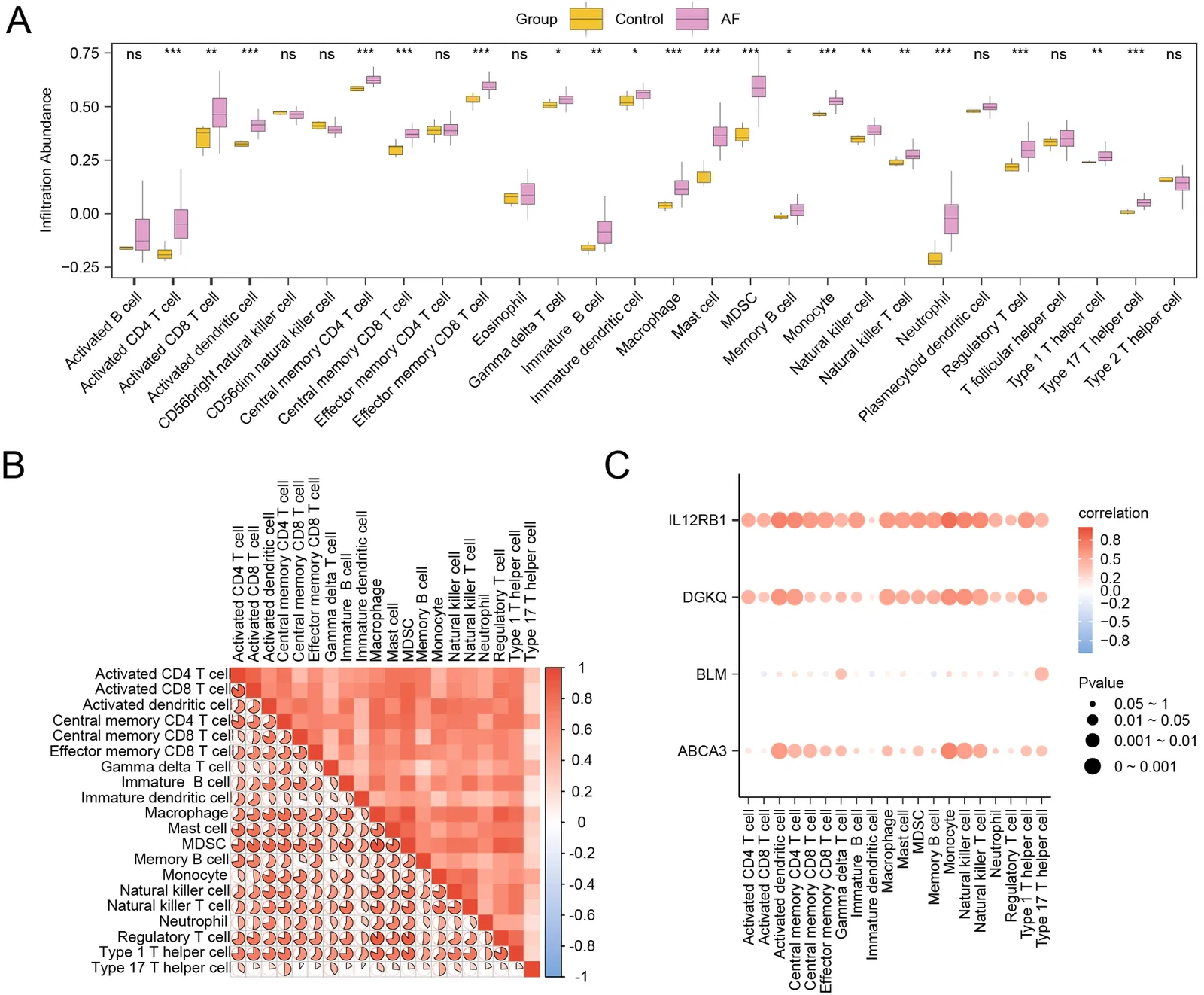
Immune infiltration analysis based on ssGSEA algorithm. **(A)** Box plot illustrating differences in immune cell infiltration abundance between the control and AF groups; **(B)** Heatmap depicting the correlation of immune cell infiltration abundance; **(C)** Bubble chart showing the correlation between key genes and immune cell infiltration abundance. ssGSEA: single-sample gene set enrichment analysis; AF: atrial fibrillation. ns indicates *p*-value ≥ 0.05, not statistically significant; * indicates *p*-value < 0.05, statistically significant; ** indicates *p*-value < 0.01, highly statistically significant; *** indicates *p*-value < 0.001, extremely statistically significant. Yellow represents the control group, and pink represents the AF group. Red denotes a positive correlation, blue denotes a negative correlation, with the color intensity indicating the strength of the correlation.

Immune infiltration in the GSE41177 atrial-tissue dataset was estimated using ssGSEA across 28 immune cell types. Boxplot analysis showed significant between-group differences in 20 immune cell populations (Figure 9A). Correlation analysis of these 20 cell types showed predominantly positive co-infiltration patterns, with the strongest correlation observed between macrophages and MDSCs (*r* = 0.923, *p* < 0.05) (Figure 9B). Correlation analysis between key genes and immune-cell abundance further showed broadly positive associations, with the strongest positive correlation observed between IL12RB1 and monocyte infiltration (*r* = 0.826, *p* < 0.05) (Figure 9C). These results suggest immune remodeling in AF atrial tissue, with IL12RB1 showing a strong positive association with monocyte infiltration within this dataset.

### scRNA-seq reveals immune cell composition shifts and Key gene expression patterns in AF

**Figure 10:**
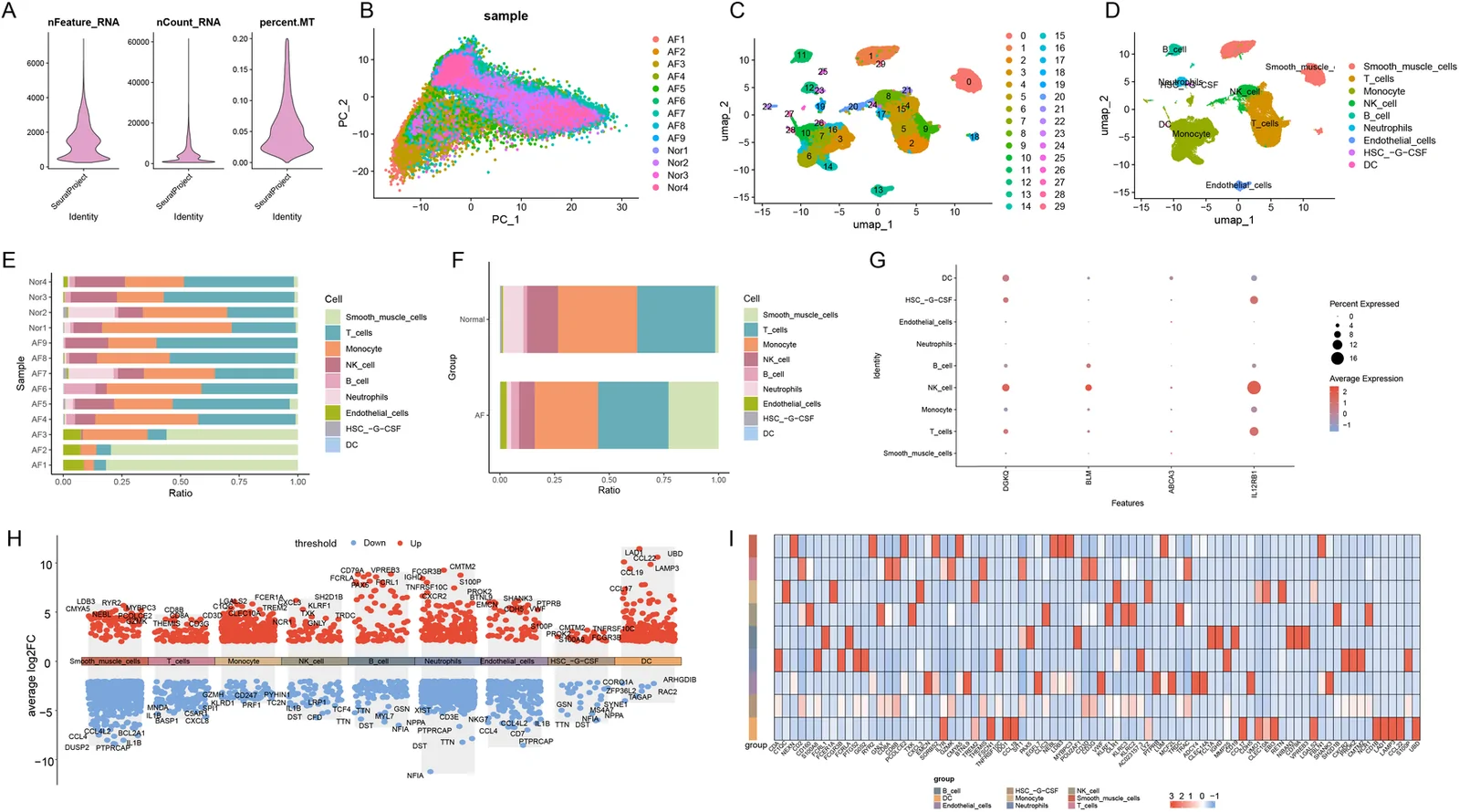
Single-Cell analysis. **(A)** Quality control violin plots for the GSE261170 dataset. **(B)** PCA visualization of cellular expression profiles across different samples. **(C)** UMAP clustering of 105,157 cells into 30 cell clusters. **(D)** Cell type annotation using the R package SingleR, classifying cells into nine cell types: smooth muscle cells, T cells, monocytes, NK cells, B cells, neutrophils, endothelial cells, hematopoietic progenitor cells (annotated as HSC-G-CSF by SingleR), and DC. **(E)** Bar plot showing the proportions of different cell types across samples. **(F)** Bar plot comparing the proportions of each cell type between the AF group and the control group. **(G)** Bubble plot illustrating the expression levels of four key genes across cell types, with darker colors indicating higher expression levels and larger bubble sizes indicating a higher proportion of cells expressing the gene within each cell type. **(H)** differential expression analysis across cell types based on scRNA-Seq data, showing the average log_2_FC of genes in the disease group relative to the control group; red dots indicate significantly upregulated genes, blue dots indicate significantly downregulated genes, and each dot represents one gene. Different cell types are demarcated by colored bars; genes were considered significantly differentially expressed at *p* < 0.05 and |log_2_FC| > 2. **(I)** Heatmap of selected significantly DEGs across cell types, with colors representing normalized gene expression levels (red, high expression; blue, low expression); the color bar on the left indicates cell type identity. AF, Atrial Fibrillation; PCA, Principal Component Analysis; UMAP, Uniform Manifold Approximation and Projection; UMI, Unique Molecular Identifier.

Single-cell RNA-seq analysis of the AF cardiac-tissue dataset GSE261170 retained 105,157 cells after quality control (Figure 10A). Principal component analysis and UMAP visualization resolved 30 clusters, which were annotated into nine cell types: smooth muscle cells, T cells, monocytes, natural killer (NK) cells, B cells, neutrophils, endothelial cells, hematopoietic progenitor cells (annotated as HSC-G-CSF), and dendritic cells (DCs) (Figures 10B–D). The relative proportions of these cell types across samples and groups are shown in Figures 10E,10F. Expression mapping of the four key genes showed that DGKQ, IL12RB1 and BLM were enriched in NK cells, whereas ABCA3 was predominantly expressed in DCs (Figure 10G). Differential expression analysis across cell populations identified cluster-enriched transcriptional features, shown by the cell-type marker volcano plot and heatmap (Figures 10H,10I). In summary, single-cell analysis indicated altered immune-cell composition in AF cardiac tissue and cell-type-preferential expression patterns of the key genes.

### *In vitro* H/R model supports that IL12RB1 knockdown suppressed NF-*κ*B activation and attenuated inflammatory responses

**Figure 11:**
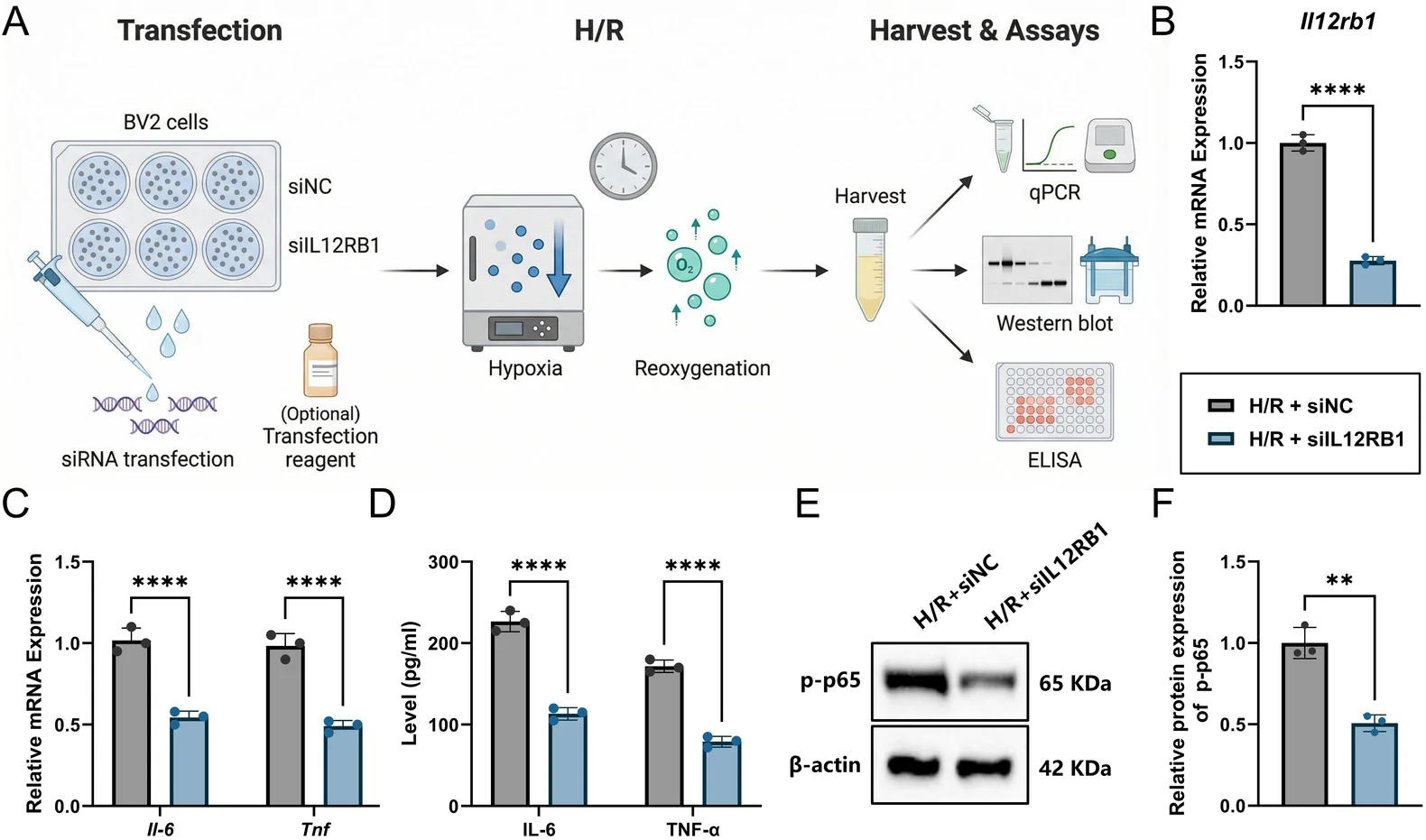
IL12RB1 knockdown suppressed NF-*κ*B activation and reduced Inflammatory Cytokine Expression and Secretion in the BV2 Cell H/R Model. **(A)** Schematic of the experimental workflow: BV2 cells were transfected with siNC or siIL12RB1, followed by H/R treatment, after which cells and culture supernatants were collected for qPCR, Western blot, and ELISA analyses. **(B)** Relative Il12rb1 mRNA expression measured by qPCR to verify knockdown efficiency. **(C)** Relative mRNA expression levels of inflammation-related genes Il-6 and Tnf measured by qPCR. **(D)** Protein levels of IL-6 and TNF-α in cell culture supernatants measured by ELISA (pg/mL). **(E)** Representative Western blot images. **(F)** Densitometric quantification of p-p65 bands. Data are presented as mean ± standard deviation, with *n* = 3 independent experiments; between-group comparisons were performed using two-tailed *t*-tests. Significance levels: ***p < 0.001, ****p < 0.0001.

To further investigate the relationship between IL12RB1 and inflammatory signaling under hypoxic stress, BV2 microglial cells were subjected to H/R and transfected with siRNA targeting Il12rb1 (Figure 11A). Compared with the H/R + siNC group, the H/R + siIL12RB1 group showed markedly reduced Il12rb1 mRNA expression (*p* < 0.0001), indicating successful knockdown (Figure 11B). Knockdown of IL12RB1 also significantly decreased the mRNA expression of Il-6 and Tnf (both *p* < 0.0001) (Figure 11C), accompanied by reduced secretion of IL-6 and TNF-α (both *p* < 0.0001) (Figure 11D). In addition, western blot analysis showed that p-p65 protein expression was reduced after IL12RB1 knockdown, which was further confirmed by densitometric analysis (*p* < 0.01) (Figures 11E,F). Together, these findings provide preliminary functional evidence that IL12RB1 may contribute to inflammatory amplification under hypoxic stress conditions, consistent with the immune-inflammatory signals observed in the AF cohort.

## Discussion

AMI markedly increased the risk of ventricular arrhythmias, yet the mechanisms by which ischemic injury translated into arrhythmia susceptibility remained incompletely defined. Using a PVN-centered multi-omics and genetic integration framework, this study showed that AMI was associated with increased arrhythmia burden, glial activation, and NF-*κ*B signaling in the PVN, together with broad transcriptomic and metabolomic remodeling. Integrative analyses further prioritized direct bilirubin and DGKQ, BLM, ABCA3, and IL12RB1 as candidate molecules linked to ventricular arrhythmia susceptibility. Independent AF cohorts showed that the molecular context surrounding these candidates was likewise characterized by inflammatory, hypoxic, and immune-remodeling features. Collectively, these findings suggested that post-AMI arrhythmia susceptibility might involve inflammatory and immune processes operating across central autonomic regulation and peripheral cardiac remodeling.

The animal phenotyping results were broadly consistent with previous studies implicating central neuroinflammatory activation in the pathophysiology of post-infarction arrhythmia. Experimental work has shown that myocardial infarction activates inflammatory signaling in the PVN, including the TLR4/MyD88/NF-*κ*B pathway, and that PVN-directed interventions can attenuate post-infarction ventricular arrhythmias and sympathetic activation (34, 35). Central microglial activation has also been implicated in post-MI sympathetic dysregulation and cardiac remodeling (36). In line with these observations, this study found increased Iba1, GFAP, and p-p65 signals in the PVN after AMI, together with increased PVC and VT burden. These findings placed PVN inflammatory activation in parallel with the development of post-infarction arrhythmia phenotypes. By incorporating PVN transcriptomics, metabolomics, and MR/SMR-based prioritization, this study extended previous work at the molecular level.

The human AF datasets provided complementary evidence from the peripheral tissue perspective. Previous studies have shown that AF atrial tissue exhibits immune remodeling, inflammatory activation, and stress-response transcriptional programs, with recent single-cell analyses further indicating marked cell-type specificity across cardiac cell compartments (22, 37). Consistent with this framework, analyses of GSE41177 and GSE261170 showed dysregulation of DGKQ, BLM, ABCA3, and IL12RB1, while GSEA, immune infiltration, and single-cell analyses highlighted inflammatory, hypoxic, and immune-remodeling features. Because these datasets were derived from human atrial tissue, their main value lay in showing that the inflammatory and immune-related molecular background linked to the prioritized candidates was not restricted to the PVN context, but was also identifiable in an independent peripheral arrhythmia setting. This supported the possibility that AF and VA after AMI might share elements of an inflammatory/immune regulatory background, without establishing a direct causal connection between central and peripheral compartments.

Taken together with prior literature, the present data supported a mechanistic framework centered on inflammatory amplification and autonomic dysregulation. AMI-induced ischemic injury was accompanied by systemic inflammatory and oxidative stress signals that aligned with PVN glial activation and NF-*κ*B upregulation, changes that may contribute to sympathetic overactivity and thereby increase arrhythmia susceptibility. Recent work has increasingly implicated neuroimmune mechanisms in post-MI arrhythmogenesis, involving not only central autonomic nuclei but also extracardiac sympathetic structures (11, 38). Within this framework, the bilirubin and IL12RB1 findings were particularly notable. Bilirubin has been regarded as an endogenous antioxidant and anti-inflammatory factor, although its protective significance in cardiovascular disease appears to depend on disease context and population characteristics (39, 40). In this study, MR suggested an inverse association between direct bilirubin and VA risk, and bilirubin supplementation reduced arrhythmia burden together with PVN inflammatory signaling. In parallel, Il12rb1 knockdown in BV2 cells reduced p-p65, IL-6, and TNF-α under H/R conditions, supporting the involvement of this pathway in inflammatory amplification during hypoxic stress. These observations collectively supported a role for inflammation- and NF-*κ*B-related signaling in post-AMI arrhythmia susceptibility and provided a basis for further mechanistic study of the prioritized candidates.

The findings also suggested potential translational relevance. Direct bilirubin and the prioritized genes, particularly IL12RB1, may warrant further evaluation as candidate biomarkers or therapeutic entry points. At present, however, the evidence remained insufficient for direct clinical application. The ROC results in GSE41177 primarily reflected within-cohort discrimination in a small atrial-tissue dataset, and the AF analyses did not establish the utility of these candidates in accessible biospecimens such as peripheral blood. Recent studies have likewise emphasized that inflammation-related markers in AF and post-MI arrhythmia remain of interest but require further validation before routine clinical use (41, 42).

Benchmarking against established arrhythmia and cardiovascular genetic frameworks is important for interpreting these candidates. Canonical ion-channel and conduction genes such as SCN5A, KCNH2, HCN4, KCNQ1, KCNE1, KCNE2, RYR2, CACNA1C, and GJA5 (43–47; Table 15) represent well-characterized arrhythmia-related loci, whereas the candidates prioritized here emerged from a PVN-centered multi-omics, MR/SMR, and AF-cohort workflow. Therefore, DGKQ, BLM, ABCA3, and IL12RB1 should be viewed as indirect or context-specific prioritized candidates rather than replacements for established AMI-, VA-, or AF-related genes.

**Table 15:** Established arrhythmia-related genes and representative cardiovascular/arrhythmia GWAS loci used for benchmarking the prioritized candidates.

**Gene/locus**	**Established arrhythmia or cardiovascular genetic context**	**Benchmark role**	**Relationship to the present candidates**
SCN5A	Cardiac sodium-channel/conduction gene linked to inherited arrhythmia syndromes and conduction phenotypes.	Canonical ion-channel/conduction reference gene for ventricular arrhythmia and inherited arrhythmia interpretation.	Established comparator; not one of the PVN-centered MR/SMR-prioritized candidates.
KCNH2	Potassium-channel gene classically linked to repolarization disorders such as long-QT syndrome and ventricular arrhythmia risk.	Canonical repolarization/channelopathy reference gene.	Established comparator; used to contextualize rather than replace DGKQ, BLM, ABCA3, and IL12RB1.
KCNQ1	Potassium-channel gene linked to inherited long-QT syndrome and arrhythmia susceptibility.	Canonical inherited arrhythmia/channelopathy reference gene.	Established comparator for interpreting whether newly prioritized candidates are canonical or context-specific.
HCN4	Pacemaker-channel gene associated with sinus-node/conduction phenotypes and arrhythmia susceptibility.	Reference gene for conduction-system and rhythm-regulation biology.	Provides established rhythm-gene background for comparison with PVN-centered candidates.
RYR2	Calcium-handling gene linked to catecholaminergic polymorphic ventricular tachycardia and ventricular arrhythmia/SCD risk.	Canonical calcium-release/ventricular-arrhythmia reference gene.	Established comparator; mechanistically distinct from the prioritized inflammatory/omics candidates.
CACNA1C	L-type calcium-channel gene implicated in inherited arrhythmia syndromes and cardiac electrophysiology.	Canonical calcium-channel/electrophysiology reference gene.	Used as an established electrophysiology benchmark.
GJA5	Connexin-40/gap-junction gene implicated in atrial conduction and atrial fibrillation susceptibility.	Atrial conduction/AF-related reference gene.	Provides AF-related comparator for evaluating the external AF-cohort signals.
PITX2, ZFHX3, KCNN3, SCN10A	Representative GWAS-supported AF/conduction loci reported in large-scale AF genetic studies.	GWAS-validated atrial arrhythmia/genetic-risk framework.	Shows that the present candidates are not being presented as established GWAS loci.
9p21/CDKN2A-CDKN2B, LDLR, PCSK9, LPA, SORT1, APOB	Representative CAD/AMI GWAS-supported cardiovascular risk loci and lipid/atherosclerosis-related genes reported in large-scale coronary artery disease genetic studies (46, 47).	AMI/CAD cardiovascular genetic-risk benchmark.	Completes the cardiovascular/AMI benchmarking framework requested by the reviewer and distinguishes established CAD/AMI risk loci from the present PVN-centered candidates.
DGKQ, BLM, ABCA3, IL12RB1	Candidates prioritized in the present PVN-centered multi-omics, MR/SMR, and external AF-cohort workflow.	Study-specific prioritized candidates.	Interpreted as indirect or context-specific candidates requiring further mechanistic validation, not replacements for canonical arrhythmia genes/loci.

The table summarizes representative established arrhythmia-related genes/loci from authoritative guideline and large-scale genetic literature (43–47) and contrasts them with the present PVN-centered MR/SMR-prioritized candidates. AF, atrial fibrillation; GWAS, genome-wide association study; MR, Mendelian randomization; PVN, paraventricular nucleus; SCD, sudden cardiac death; SMR, summary-data-based Mendelian randomization.

Several limitations should be acknowledged. First, this study integrated mouse PVN multi-omics with human atrial-tissue datasets, and the scope of inference across species and tissues was therefore inherently limited. Second, the MR/SMR analyses relied on blood-derived eQTL instruments, which strengthened candidate prioritization but did not establish PVN-specific regulatory effects. Third, colocalization analyses did not identify moderate or strong shared causal signals at the prioritized loci, indicating that the current genetic evidence was more consistent with convergent prioritization than definitive causal resolution. Fourth, functional validation remained restricted: only IL12RB1 underwent functional validation, whereas DGKQ, BLM, and ABCA3 remain computationally prioritized candidates requiring further mechanistic investigation. In addition, bilirubin received *in vivo* pharmacological support, but it should still be interpreted as a candidate protective metabolite rather than definitive clinical evidence. Fifth, BV2 cells captured only part of PVN microglial biology and could not recapitulate the full hypothalamic microenvironment. Finally, the public AF cohorts were limited in size and clinically heterogeneous, which may have affected the robustness and generalizability of some findings.

Future studies should establish a more direct causal chain linking PVN remodeling to arrhythmia phenotypes after AMI. Region-specific genetic or pharmacological manipulation of the PVN, combined with measurements of sympathetic output, catecholamine signaling, and electrophysiological susceptibility, would help define the contribution of PVN inflammatory pathways to post-infarction arrhythmogenesis. It will also be important to identify the PVN cell populations responsive to AMI and bilirubin, for example through single-cell and spatial transcriptomic approaches in hypothalamic tissue. On the translational side, prospective clinical studies in AMI should determine whether bilirubin-related metabolic features or candidate-gene readouts are associated with subsequent VA or AF risk.

## Conclusion

This study showed that inflammatory activation, glial reactivity, and enhanced NF-*κ*B signaling in the PVN after AMI were accompanied by increased susceptibility to ventricular arrhythmias, suggesting that central molecular remodeling may contribute to the development of the post-infarction arrhythmic phenotype. Integrating multi-omics, genetic analyses, and an external AF cohort, direct bilirubin as well as DGKQ, BLM, ABCA3, and IL12RB1 were prioritized as candidate molecules warranting further investigation. Among these, bilirubin intervention and IL12RB1 knockdown provided supportive *in vivo* and *in vitro* evidence, respectively, for inflammation-related mechanisms. Overall, these findings support a PVN-centered framework in which metabolic, inflammatory, immune, and neural regulatory processes may jointly influence arrhythmia susceptibility after AMI, and they offer clues for subsequent mechanistic studies and translational validation.

## Data Availability

The original contributions presented in the study are included in the article/Supplementary Material, further inquiries can be directed to the corresponding author.

## Supplementary material

Supplementary Figure S1 Schematic overview of the experimental design for AMI mouse model and brain tissue collection.

## Abbreviations

AF: Atrial Fibrillation
AFL: Atrial Flutter
AMI: Acute Myocardial Infarction
ANOVA: Analysis of Variance
AUC: Area Under the Curve
BH: Benjamini-Hochberg
BP: Biological Processes
CA: Cardiac Arrhythmias
CC: Cellular Components
CI: Confidence Interval
cTnI: Cardiac Troponin I
DC: Dendritic Cells
DEGs: Differentially Expressed Genes
DEMs: Differentially Expressed Metabolites
DSigDB: Drug Signature Database
eQTL: Expression Quantitative Trait Loci
ELISA: Enzyme-Linked Immunosorbent Assay
FC: Fold Change
FDR: False Discovery Rate
GO: Gene Ontology
GSEA: Gene Set Enrichment Analysis
GWAS: Genome-Wide Association Studies
HSC-G-CSF: Granulocyte Colony-Stimulating Factor–Mobilized Hematopoietic Progenitor Cells (SingleR Annotation)
IVs: Instrumental Variables
IVW: Inverse Variance Weighted
KEGG: Kyoto Encyclopedia of Genes and Genomes
LAD: Left Anterior Descending Coronary Artery
LD: Linkage Disequilibrium
MDSC: Myeloid-Derived Suppressor Cells
MF: Molecular Functions
MR: Mendelian Randomization
NES: Normalized Enrichment Score
NK: Natural Killer
OR: Odds Ratio
PCA: Principal Component Analysis
PDB: Protein Data Bank
pLDDT: Predicted Local Distance Difference Test
PVN: Paraventricular Nucleus
ROC: Receiver Operating Characteristic
SCD: Sudden Cardiac Death
scDEGs: Single-Cell Differentially Expressed Genes
scRNA-seq: Single-Cell RNA Sequencing
SEM: Standard Error of the Mean
SG: Stellate Ganglion
SMR: Summary-Data-Based Mendelian randomization
SNP: Single Nucleotide Polymorphism
SNS: Sympathetic Nervous System
ssGSEA: Single-Sample Gene Set Enrichment Analysis
TTC: 2,3,5-Triphenyltetrazolium Chloride
TPR: True Positive Rate
UMAP: Uniform Manifold Approximation and Projection
VA: Ventricular Arrhythmia
VIP: Variable Importance in Projection.
